# Artificial Organelles with Orthogonal‐Responsive Membranes for Protocell Systems: Probing the Intrinsic and Sequential Docking and Diffusion of Cargo into Two Coexisting Avidin–Polymersomes

**DOI:** 10.1002/advs.202004263

**Published:** 2021-04-07

**Authors:** Xueyi Wang, Silvia Moreno, Susanne Boye, Peng Wang, Xiaoling Liu, Albena Lederer, Brigitte Voit, Dietmar Appelhans

**Affiliations:** ^1^ Leibniz‐Institut für Polymerforschung Dresden e.V. Hohe Straße 6 Dresden 01069 Germany; ^2^ Organic Chemistry of Polymers Technische Universität Dresden Dresden 01062 Germany; ^3^ College of Polymer Science and Engineering Sichuan University Chengdu 610065 P. R. China; ^4^ Department of Chemistry and Polymer Science Stellenbosch University Matieland 7602 South Africa

**Keywords:** artificial organelles, avidin, biohybrid structures, intrinsic cargo diffusion, pH‐responsive polymersomes

## Abstract

The challenge of effective integration and use of artificial organelles with orthogonal‐responsive membranes and their communication in eukaryotic protocells is to understand the intrinsic membrane characteristics. Here, a novel photo‐crosslinked and pH‐responsive polymersome (Psome B) with 2‐(*N*,*N*′‐diisopropylamino)ethyl units in the membrane and its respective Avidin‐Psome B hybrids, are reported as good candidates for artificial organelles. Biotinylated (macro)molecules are able to dock and diffuse into Avidin‐Psome B to carry out biological activity in a pH‐ and size‐dependent manner. Combined with another polymersome (Psome A) with 2‐(*N*,*N*′‐diethylamino)ethyl units in the membrane, two different pH‐responsive polymersomes for mimicking different organelles in one protocell system are reported. The different intrinsic docking and diffusion processes of cargo (macro)molecules through the membranes of coexisting Psome A and B are pH‐dependent as confirmed using pH titration–dynamic light scattering (DLS). Psome A and B show separated “open”, “closing/opening”, and “closed” states at various pH ranges with different membrane permeability. The results pave the way for the construction of multicompartmentalized protocells with controlled communications between different artificial organelles.

## Graphical abstract

For studying orthogonal‐responsive membranes in eukaryotic protocells, artificial organelles are constructed by two different pH‐stable and pH‐responsive avidin–polymersome biohybrid structures. Different pH‐dependent “open”, “closing/opening”, and “closed” membrane states of the two coexisting biohybrid structures pave the way to different intrinsic and sequential docking and diffusion processes of cargo (macro)molecules in a pH‐ and size‐dependent manner.

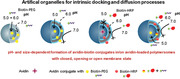

## 1 Introduction

In nature, eukaryotic cells have a complex and multicompartmentalized architecture consisting of organelles with various sizes <1 µm (e.g., mitochondria, lysosomes and Golgi apparatus), entrapping specific enzymes and other biomolecules inside their own membranes to achieve differently biological activities. The biological membranes of cells and organelles play a key role in living organisms by providing spatially isolated environment for enzymatic reactions and transmembrane transport for the communication between different compartments.^[^ ^1^ ^]^

For mimicking abovementioned cellular (multi)compartments (e.g., artificial organelles^[^ ^2^, ^3^, ^4^, ^5^ ^]^ and protocells),^[^ ^6^, ^7^, ^8^ ^]^ different synthetic vesicles (e.g., liposomes,^[^ ^9^, ^10^, ^11^ ^]^ hollow capsules,^[^ ^12^, ^13^, ^14^ ^]^ polymersomes^[^ ^15^, ^16^, ^17^, ^18^ ^]^ and proteinosomes^[^ ^5^, ^6^, ^19^, ^20^, ^21^, ^22^ ^]^) and their multicompartments^[^ ^23^, ^24^, ^25^, ^26^ ^]^ have been designed. Increasing the complexity and diversity of compartments is a crucial issue for mimicking iterative and/or feedback‐controlled processes of and between cellular compartments,^[^ ^27^, ^28^, ^29^ ^]^ for mimicking dynamic self‐assembly and disassembly within protocells,^[^ ^7^, ^20^ ^]^ and for mimicking fusion of cellular compartments.^[^ ^19^, ^30^ ^]^ Especially, the fluidity as well as multiple and different responsiveness of polymeric or lipidic membranes in cell mimics is responsible for the success in the construction of hierarchical and communicating cell compartments,^[^ ^24^ ^]^ as well as for other membrane characteristics in diverse cell mimics without and with membrane proteins and protocells for exchanging cargo (macro)molecules.^[^ ^26^, ^29^, ^31^, ^32^, ^33^, ^34^, ^35^, ^36^, ^37^, ^38^, ^39^, ^40^ ^]^

In eukaryotic cells, the pH value within organelles is different and partially outlines a gradient behavior (e.g., pH 7.2 for nucleus, pH range from 6.0 to 6.7 for Golgi network, and pH 4.7 for lysosome).^[^ ^41^ ^]^ Thus, pH‐responsive vesicles,^[^ ^29^, ^42^, ^43^, ^44^, ^45^, ^46^, ^47^, ^48^, ^49^ ^]^ used as cell mimics, are able to tune the release of cargo^[^ ^42^, ^43^, ^45^, ^46^, ^47^ ^]^ and the switch off and on enzymatic reactions.^[^ ^29^, ^48^, ^49^ ^]^

Until now, scientific reports have been focused on pH‐responsive vesicles for one kind of pH‐responsive artificial organelle, but not simultaneously on two or more types of artificial organelles showing different pH‐responsiveness for opening and closing their membranes. The still insufficient understanding of pH homeostasis in eukaryotic cells motivated us, first, to establish an artificial organelle system with different pH‐responsiveness of the membranes. For establishing communicating artificial organelles and for mimicking cellular functions and homeostasis processes, the following approaches were selected: i) preparing polymersomes with different membrane permeability responding to one specific pH value; ii) mimicking organelles for controlled uptake of cargo (= crossing artificial organelle membrane from outside to inside); iii) integration of avidin in the membrane and lumen and on the surface of artificial organelles to use it as docking platform; and iv) understanding the membrane permeability of cargo (= diffusion pathways) under different conditions.

Polymersomes, constructed by the self‐assembly of amphiphilic block copolymers, are the synthetic counterpart of the natural liposomes and consist of polymeric (bilayer) membrane and aqueous lumen.^[^ ^50^, ^51^, ^52^, ^53^ ^]^ Thus, they are promising candidates for the design and construction of artificial organelles because of their high physicochemical stability and chemical versatility.^[^ ^15^, ^16^, ^54^, ^55^ ^]^ To modulate the membrane permeability of polymersomes as artificial organelles^[^ ^56^, ^57^ ^]^ for preferentially smaller molecules,^[^ ^58^, ^59^ ^]^ different stimuli‐responsive moieties into the hydrophobic part of amphiphilic block copolymer have been introduced, for example, responding on light,^[^ ^28^, ^60^ ^]^ redox,^[^ ^61^, ^62^ ^]^ temperature,^[^ ^63^, ^64^ ^]^ and pH.^[^ ^27^, ^65^, ^66^, ^67^, ^68^, ^69^ ^]^ Because of the physiological pH gradients in the body, pH‐responsive and (photo‐)crosslinked polymersomes^[^ ^27^, ^65^, ^66^, ^67^, ^68^, ^69^, ^70^, ^71^, ^72^, ^73^ ^]^ are ideal models for artificial organelles in the present study. Previously, 2‐(*N*,*N*′‐diethylamino)ethyl methacrylate (DEAEMA) and photo‐crosslinkers (e.g., 4‐(3,4‐dimethylmaleimidio)butyl methacrylate (DMIBMA)^[^ ^74^ ^]^) have been integrated into the hydrophobic part of block copolymers, possessing a hydrophilic poly(ethylene glycol) (PEG) tail, for the fabrication of pH‐responsive polymersomes (called Psome A).^[^ ^66^, ^67^, ^68^, ^70^, ^71^, ^75^ ^]^ As a result, the membrane in Psome A becomes permeable towards molecules with uni‐ and bidirectional cargo transport at a specific pH value.^[^ ^66^, ^69^, ^70^, ^71^, ^75^ ^]^

In a recent study, the abovementioned approaches (steps i–iv) of a pH‐responsive avidin‐loaded Psome A (Avidin‐Psome A) have been validated successfully.^[^ ^75^ ^]^ Photo‐crosslinked, pH‐responsive Psome A^[^ ^66^, ^67^, ^68^, ^70^, ^71^, ^75^ ^]^ outlines an excellent and reversible swelling (pH ≤ 7)/shrinking (pH > 7) behavior. This results in controllable membrane permeability for cargo uptake with dimension up to 10 nm and more,^[^ ^70^, ^71^ ^]^ making them good candidates as artificial organelles. In 10 × 10^−3^ m NaCl solution, pH* of Psome A (turning point of pH dependent size transition, showing half power of swelling)^[^ ^68^ ^]^ is about 6.5, while pH^0^ (starting point of swelling) of Psome A is 7.0. Both key characteristics can be shifted to higher pH values when increasing salt and/or buffer concentration.^[^ ^68^ ^]^ In line with this, Avidin‐Psome A possesses the same key characteristics for pH* and pH^0^ as found for pure Psome A validated under the same conditions.^[^ ^75^ ^]^ Moreover, Avidin‐Psome A demonstrates a high pH stability and does not release avidin biomacromolecules at neutral and acidic pH. This allowed us to study successfully the diffusion pathways and docking processes of biotinylated cargo on collapsed and swollen Avidin‐Psome A, but also to identify the location(s) of docked cargo.^[^ ^75^ ^]^

**Figure 1:**
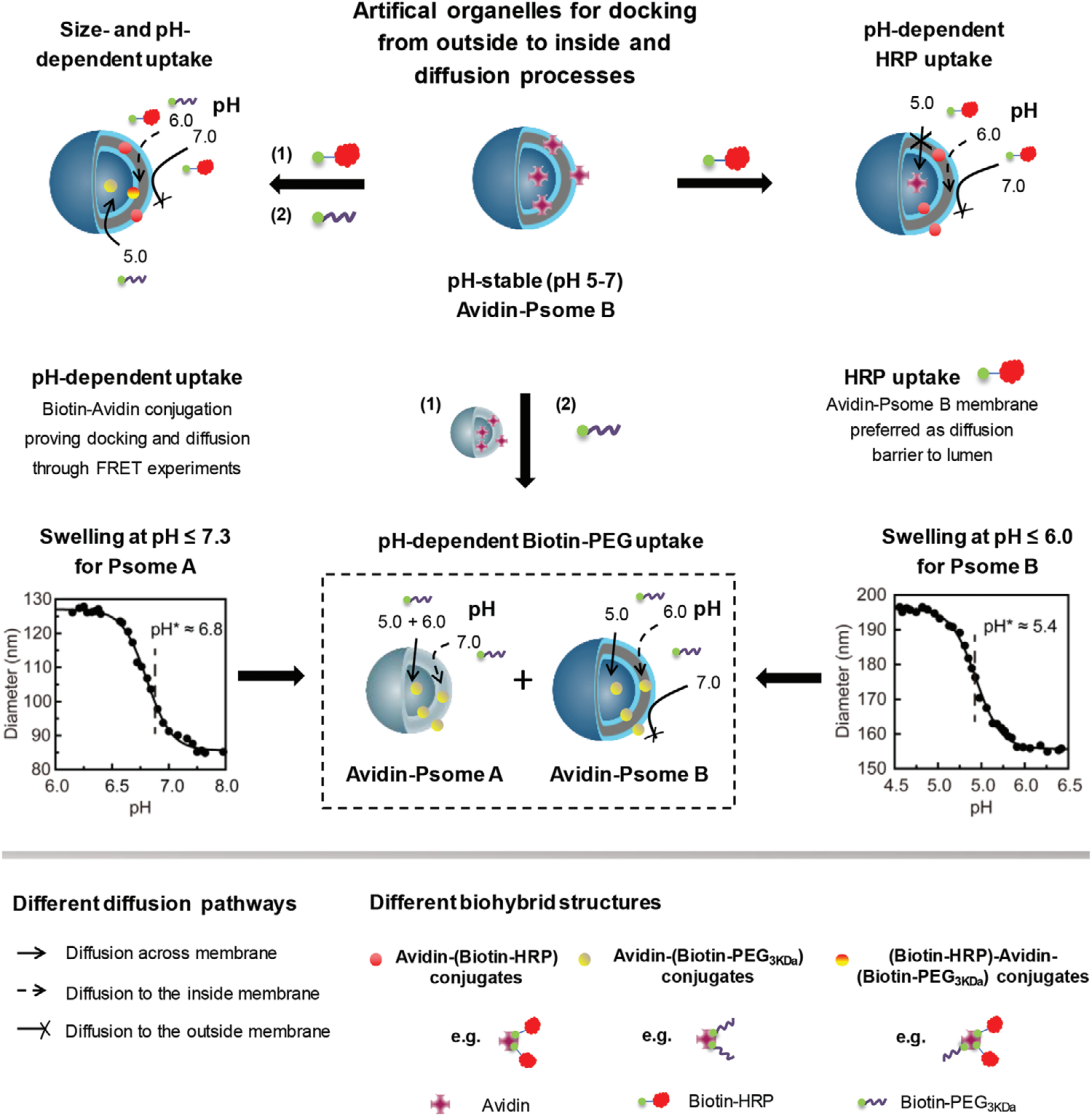
Schematic overview of versatile pH‐responsive polymersome B (Psome B) for mimicking artificial organelles due to: i) its intrinsic pH‐dependent membrane permeability, and ii) its pH‐stable non‐covalent interactions between cationic avidin and cationic Psome B membrane. Sequential uptake (= docking and diffusion) of cargo by artificial organelles is triggered by 2‐*(N*,*N*′‐diisopropylamino)ethyl units with pK_a_ = 6.0 in Psome B membrane (pH* as half power of polymersome swelling is 5.4 in 1 × 10^−3^ m PBS buffer) and 2‐(*N*,*N*′‐diethylamino)ethyl units with pK_a_ = 7.0–7.3 in polymersome A (Psome A) membrane (pH* is 6.8 in 1 × 10^−3^ m PBS buffer). Docking = undergoing avidin–biotin conjugation. Diffusion = crossing membrane from outside to inside and finally, into the lumen. Fluorescence resonance energy transfer (FRET) experiments are carried out to evidence the different states of uptake.

**Figure 2:**
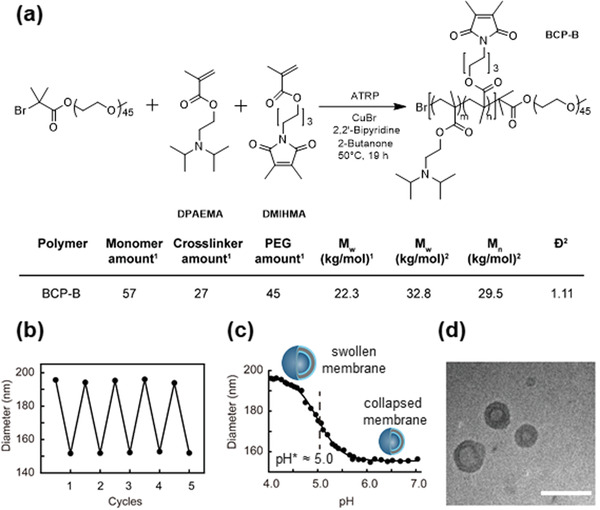
Synthesis of block copolymer B (BCP‐B) and characterization of Psome B. a) Reaction scheme, composition, molecular weight, and dispersity (*Ð*) of BCP‐B. ^1^Determined by ^1^H‐NMR spectroscopy;^2^ Determined by GPC. b) Reversible swelling/deswelling of Psome B at pH 4.0 and 7.0 in water solution at ≈10 × 10^−3^ m NaCl by DLS. c) DLS–titration data of 0.5 mg mL^−1^ Psome B in water solution at ≈10 × 10^−3^ m NaCl (• Diameter from DLS, **—** Logistic fit of the curve). d) Cryo‐TEM image of Psome B, scale bar: 200 nm. Average diameter ≈ 120.6 ± 32.3 nm and membrane thickness ≈ 26.9 ± 2.9 nm, taken from more than 100 polymeric vesicles.

**Figure 3:**
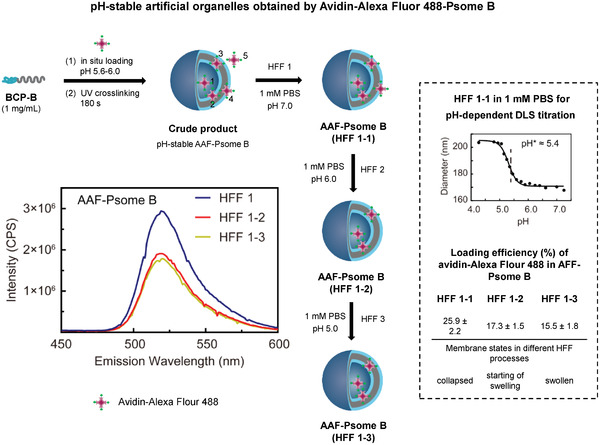
Protocol for the establishment of pH‐stable Avidin–Alexa Flour 488 loaded polymersome B (Avidin–Alexa Fluor 488–Psome B(AAF‐Psome B)) through sequential pH‐dependent shear‐force‐driven hollow‐fiber‐filtration (HFF). The avidin locations: 1 (lumen), 2 (inner hydrophilic shell of membrane), 3 (hydrophobic membrane), 4 (membrane‐integrated avidin in the outer hydrophilic shell of membrane), and 5 (free). Loading efficiency (%) of different HFF purified AAF‐Psome B samples (right bottom: HFF B1, HFF B1‐2, and HFF B1‐3) calculated from the fluorescence spectra. pH‐dependent DLS–titration of AAF‐Psome B (HFF B1) (right) for validating collapsed membrane, starting of swelling membrane, and highly swollen membrane states for AAF‐Psome B (HFF B1) during different HFF purification processes at pH 7.0, 6.0, and 5.0, respectively.

**Figure 4:**
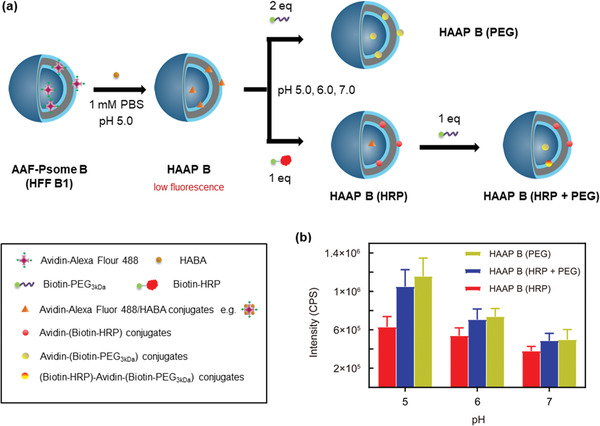
Study of pH‐ and size‐dependent cargo docking and diffusion processes of the membrane in Avidin–Alexa Fluor 488‐loaded polymersome B after HFF B1 (AAF‐Psome B) hybrid structures. HAAP B = AAF‐Psome B in the presence of excess HABA. a,b) Protocol (a) and results (b) for FRET experiments of different HAAP B samples after biotinylated cargo uptake through fluorescence measurements, leading to the formation of different Avidin–Alexa Fluor‐488/Biotin–cargo conjugates (HAAP B (HRP), HAAP B (HRP + PEG) and HAAP B (PEG)). Further details on the size‐ and pH‐dependent cargo docking and diffusion process are shown in Table S5 and Figure S20, Supporting Information.

**Figure 5:**
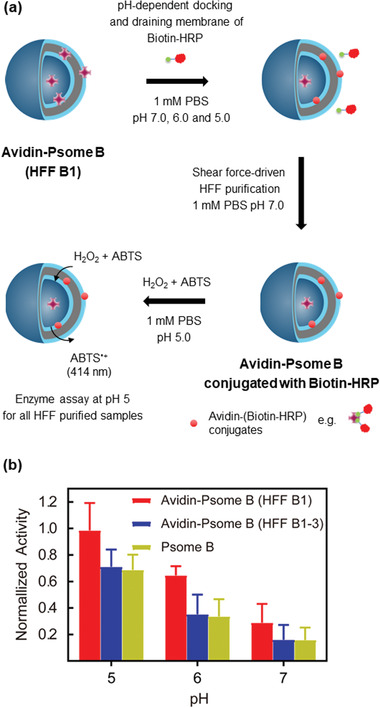
Enzyme assay for artificial organelle studied after pH‐dependent Biotin‐HRP uptake. a) Protocol for enzyme assay of Biotin‐HRP conjugated to Avidin‐Psome B (HFF B1). b) Comparing the enzymatic activity between sample Avidin‐Psome B (HFF B1) and references, pure Psome B, Avidin‐Psome B (HFF B1‐3) (Figure S21, Supporting Information).

**Figure 6:**
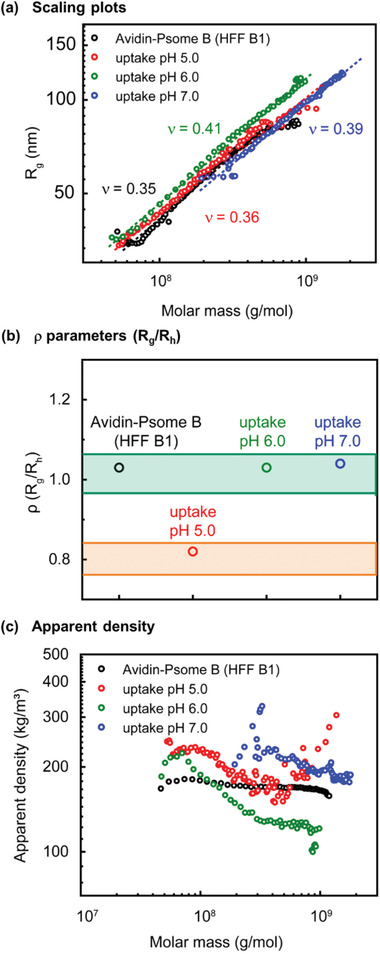
Conformational properties of Avidin‐Psome B (HFF B1) (black), loaded with Biotin‐PEG_3kDa_ at varied pH (5.0 = red; 6.0 = green, 7.0 = blue): a) scaling plots, *R*_g_ versus molar masses; (b) *ρ* parameters (*R*_g_/*R*_h_) at concentration maximum, (*ρ* parameters in the grass‐green area: vesicles are soft spheres with inhomogeneous and rough surface; *ρ* parameters in the orange‐yellow area: vesicles are ideal, hard sphere with smooth surface), and c) apparent densities versus molar masses determined by AF4‐LS.

**Figure 7:**
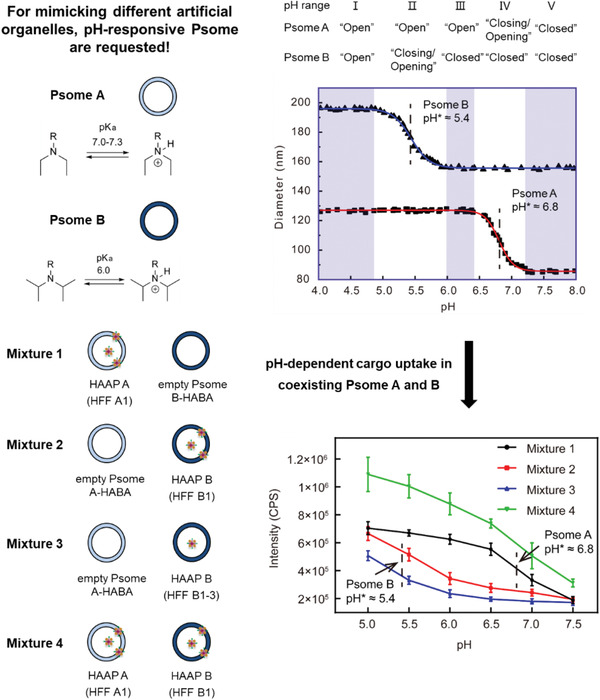
The pH‐dependent “open”, “closing/opening”, or “closed” state of pH‐responsive Psome A and B demonstrating the pH‐dependent Biotin‐PEG_3kDa_ diffusion into coexisting Psome A and B through FRET experiments. The diffusion of Biotin‐PEG_3kDa_ is triggered by the different pH^0^ (starting point of swelling) and pH* (turning point of pH‐dependent size transition). DLS–titration data of Psome A and B in 1 × 10^−3^ m PBS buffer and their different pH‐dependent “open”, “closing/opening”, or “closed” state (top). The fluorescence intensity at 518 nm of Avidin–Alexa Fluor 488 conjugate in Mixture 1 (HAAP A (HFF A1) and empty Psome B–HABA mixture; HAAP A (HFF A1) through purification of AAF‐Psome A with HFF A1 and followed by the addition of HABA (Figure S16, Supporting Information)), Mixture 2 (empty Psome A‐HABA and HAAP B (HFF B1) mixture; HAAP B (HFF B1) through purification of AAF‐Psome B with HFF B1 and followed by the addition of HABA (Figure 3)), Mixture 3 (empty Psome A‐HABA and HAAP B (HFF B1‐3) mixture; HAAP B (HFF B1‐3) through purification of AAF‐Psome B with sequential HFF B1‐3 (Figure 3) and followed by the addition of HABA), and Mixture 4 (HAAP A (HFF A1) and HAAP B (HFF B1) mixture) by adding Biotin‐PEG_3kDa_ at various pH values for carrying out FRET experiments (bottom). Mixture 5 (empty HABA‐Psome A and B mixture) as blank experiment outlines no fluorescent properties (Figure S24, Supporting Information).

For fabricating pH‐responsive artificial organelle systems (**Figure** **1**), one main goal of this study was to fabricate and characterize a second artificial organelle, Avidin‐Psome B, possessing a lower pH* (≤5.4) and a lower pH^0^ (≤6.0) than that of artificial organelle Avidin‐Psome A (Figure 1). To achieve the desired membrane characteristics, we replaced DEAEMA monomer by 2‐(*N*,*N*′‐diisopropylamino)ethyl methacrylate (DPAEMA) monomer characterized by a lower pK_a_ value than DEAEMA^[^ ^76^, ^77^, ^78^, ^79^ ^]^ to establish a novel photo‐crosslinkable amphiphilic block copolymer B (BCP‐B) for the fabrication of pH‐switchable Psome B (**Figure** **2**). With BCP‐B we were able to investigate the potential of both pH‐responsive artificial organelles, Avidin‐Psome A and B, individually and simultaneously, through the (sequential) pH‐dependent uptake of biotinylated cargo (**Figures** **3**–**7**).

For the determination of cargo locations after cargo uptake (Figure 1), fluorescence resonance energy transfer (FRET) experiments in combination with hollow filtration experiments (HFF), asymmetrical flow‐field flow fractionation (AF4) and/or enzyme assays were used. This allowed us to postulate main locations of cargo on and in final Avidin‐Psome B and to compare the uptake potential of biotinylated cargo in both artificial organelles.

## 2 Results and Discussion

### 2.1 Preparation and Characterization of Psome B

According to the idea of transmembrane transport from cytosol to different organelles, a new photo‐crosslinkable pH‐responsive block copolymer (BCP‐B) was synthesized via ATRP polymerization. BCP‐B consists of PEG in the hydrophilic block and the pH‐responsive 2‐(*N*,*N*′‐diisopropylamino)ethyl methacrylate (DPAEMA) and the UV‐active photo‐crosslinker 6‐(3,4‐dimethylmaleimidio)hexyl methacrylate (DMIHMA) in the hydrophobic block (see Figure 2a; Figure S4, Supporting Information, for details of BCP‐B fabrication). To obtain thoroughly cyclic pH‐switchable polymersomes (Psome B) based on BCP‐B (Figure 2b) with an acid‐induced swelling at pH ≤ 6 (Figure 2c), a higher degree of photo‐crosslinker DMIHMA (30 mol%) was integrated into the hydrophobic block of BCP‐B through statistical polymerization of DPAEMA and DMIHMA. BCP‐B was validated by ^1^H NMR spectroscopy and GPC (Figure 2a; Figure S5, Supporting Information). Thus, the molecular composition, molecular weight and dispersity (*Ð*) of BCP‐B were determined and are presented in Figure 2a. Due to the bulkiness of diisopropylamino part structure of DPAEMA, a C6‐spacer in the photo‐crosslinker was needed to allow the necessary cross‐linking density of the hydrophobic membrane of Psome B, obtained through the self‐assembly of BCP‐B at pH 5.6–6.0 followed up by the UV‐irradiation for at least 180 s. The results in the desired mechanical stability of Psome B for carrying out all further experiments are described here in this study. Other combinations, for example, lower molar ratio between DPAEMA and DMIHMA in the hydrophobic block and shorter time of UV irradiation, induce undesired full or partial disassembly of Psome B. Further details of this optimization are presented in Table S2, Supporting Information.

Psome B, fabricated by the pH‐switch method,^[^ ^78^ ^]^ was further analyzed by cyclic pH‐switches, pH‐dependent dynamic light scattering (DLS)–titration and cryo‐TEM study (Figure 2b–d). Psome B outlines the desired key characteristics as known from our standard Psome A,^[^ ^66^, ^67^, ^68^, ^70^, ^71^, ^75^ ^]^ which was fabricated by the self‐assembly of pH‐responsive block copolymers (BCPs) called BCP‐A consisting of PEG as the hydrophilic block, DEAEMA as the pH‐responsive part, and DMIBMA (crosslinker A) as the photo‐crosslinker in the hydrophobic block (see Figures S2, S3 and Table S1, Supporting Information, for the details of BCP‐A fabrication). Due to the lower pK_a_ of DPAEMA^[^ ^78^, ^79^ ^]^ in the membrane of Psome B, a lower pH^0^ (= starting point of swelling at pH 6, at 10 × 10^−3^ m NaCl), a lower pH* (= half power of polymersome swelling at pH 5, Figure 2c; see calculation of pH* and pH^0^ in Figure S7, Supporting Information), and also larger hydrodynamic diameters at collapsed (≈150 nm) and swollen (≈195 nm) state (Figure 2b) for Psome B are given compared to Psome A (pH* 6.5 and pH^0^ 7.0 at 10 × 10^−3^ m NaCl (Figure S8a, Supporting Information); see the Supporting Information for further details about Psome A). Cyclic pH switches of Psome B on demand at pH 7.0 (collapsed state) and 4.0 (swollen state) for 5 cycles also provide no disassembly and membrane rupture as well as no aggregation with increasing ion concentrations. From cryo‐TEM images, spherical vesicles can be assumed. This characteristic was also confirmed by asymmetrical flow‐field flow fractionation (AF4) (Figure S26, Supporting Information). The average diameter and membrane thickness of Psome B (Figure 2d) are about 121 and 27 nm, respectively, in collapsed state at pH 7.0. In opposite, results from cryo‐TEM for Psome A (Figure S9b, Supporting Information) outline diameters of about 80 nm and membrane thickness of about 16 nm. Overall, the substitution of DEAEMA by DPAEMA in a photo‐crosslinkable BCP allowed us to establish novel pH‐switchable Psome B with lower pH* and pH^0^ than that of Psome A (Figure S8, Supporting Information). Comparison of characteristics between Psome A and B is presented in Table S3, Supporting Information.

For elucidating the effect of different salt concentrations, we studied pH* and pH^0^ of Psome B under 1 and 10 × 10^−3^ m PBS buffer. Results of pH‐dependent DLS–titrations are presented in Figure S10a,b, Supporting Information. Thus, pH* of Psome B is 5.4 for 1 × 10^−3^ m PBS and 6.0 for 10 × 10^−3^ m PBS, respectively. pH^0^ (pH = 6.0) in 1 × 10^−3^ m PBS is similar as found for Psome B in 10 × 10^−3^ m NaCl (Figure 2c), while in 10 × 10^−3^ m PBS pH^0^ is shifted to pH 6.5. Thus, higher salt concentrations increase the pH* and pH^0^ (Figure S10c, Supporting Information). This is in accordance with previously published results of Psome A.^[^ ^68^ ^]^ In addition, the pH‐dependent zeta potential (ZP) of Psome B in 1 × 10^−3^ m PBS buffer was also measured. The results (Figure S10d, Supporting Information) lead to the conclusion that at around pH 7.2 the ZP of Psome B reaches 0 mV in comparison to Psome A (above pH 8.0) (Figure S8d, Supporting Information). This further confirms that DPAEMA is protonated at a lower pH than DEAEMA. This also implies that surface charge with 0 mV at 1 × 10^−3^ m PBS is different from pH^0^ for Psome B (Figure S10, Supporting Information). Thus, the next step was to validate their characteristics of membrane transport for elucidating their potential as artificial organelles under defined ion concentrations (Figure 1).

### 2.2 Fabrication of Avidin‐Psome B and Avidin's Location

The biological functions of a cell and their organelles are orchestrated by the interplay of peptides, proteins, and peptide/protein complexes in nano/micrometer‐sized dimensions. To obtain simplified functions of artificial organelles by Psome B, protein uptake through swollen membranes is a challenging step (Figure 1). This will provide the possibility to mimic enzymatic metabolism inside or within the membrane of artificial organelles. Thus, pH‐stable avidin‐loaded Psome B hybrid structures (Avidin–Alexa Flour 488–Psome B = AAF‐Psome B) were established (Figure 3) to study their cargo diffusion processes from outside to inside, (sequential) docking processes (Figure 4), and enzymatic metabolism (Figure 5). This includes the localization of avidin in AAF‐Psome B (locations 1–4, Figure 3) which should be stable when carrying out experiment series at pH 7.0, 6.0, and 5.0 (Figures 4, 5, and 7).

The fabrication of pH‐stable AAF‐Psome B (HFF B1, after first HFF purification against 1 × 10^−3^ m PBS at pH 7.0) is presented in Figure 3 and Figure S11, Supporting Information. The results of pH‐dependent DLS–titration of HFF B1 (Figure 3) undoubtedly show that pH* and pH^0^ for Psome B outline the same values as found for pure Psome B (Figure 2b). HFF B1 shows negligible sequential release of avidin–Alexa Flour 488 conjugates from HFF B1 after sequential dialysis (see Figure S14a,d, Supporting Information, for details). Thus, AAF‐Psome B (HFF B1) fulfills the required key characteristics—abovementioned—for the next FRET experiments (Figures 4, 5, and 7).

To better understand the results of the FRET experiment (presented below) for the membrane diffusion, docking, and sequential uptake processes (Figures 4, 5, and 7), the study of different locations of avidin within Psome B is essential. Thus, we determined the loading efficiency of avidin–Alexa Flour 488 conjugates (AAF‐488) in Psome B after sequential HFF steps against 1 × 10^−3^ m PBS at pH 6.0 (HFF B1‐2, see Supporting Information for details) and 5.0 (HFF B1‐3, see Supporting Information for details), separately. Avidin can be taken up in location 1 = lumen, 2 = inner hydrophilic shell of membrane, 3 = hydrophobic membrane, and 4 = integrated in the outer hydrophilic shell of membrane. The protocol and results of HFF B1–HFF B1‐3 from fluorescence study are shown in Figure 3.

After HFF B1 purification at pH 7, the loading efficiency of AAF‐488 in HFF B1 is around 25.9 ± 2.2%. With this we conclude that free AAF‐488 (location 5) and weakly attached AAF‐488 at the outer hydrophilic shell of membrane (location 4) are removed, while the residual AAF‐488 are randomly distributed at different locations (1–4). This is supported by the collapsed state of Psome B membrane for HFF B1 at pH 7.0 in 1 × 10^−3^ m PBS buffer (Figure 3, top right) with no obvious AAF‐488 release from the inner part.

Finally, we obtained pH‐stable Avidin–Alexa Flour 488‐Psome B (HFF B1‐2 and B1‐3) under applied shear‐force driven HFF purification (Figure 3). HFF B1‐2 and HFF B1‐3 for AAF‐Psome B show avidin mainly positioned in locations 1–3 compared to HFF B1 (Figure 3). The results suggest higher membrane‐anchored avidin in Avidin‐Psome B in comparison to Avidin‐Psome A (Figure S16, Supporting Information),^[^ ^75^ ^]^ which might be attributed to the large membrane thickness. Most of the membrane‐anchored avidin in Avidin‐Psome B (location 4) is removed by HFF B2 and B3 processes, which is also confirmed later. It is noteworthy that HFF B1‐3 of AAF‐Psome B (Figure 3) still shows loading efficiency (15.5%). Thus, assuming, avidin is located in the lumen and inner membrane. Moreover, all samples fulfill the desired key characteristic of “non‐releasing avidin” after sequential dialysis at different pH (Figure S14, Supporting Information) due to a combination of non‐covalent interactions, kind of entrapment of avidin in membrane, and membrane as diffusion barrier for avidin from inside (= lumen) to outside over a broader pH range (pH 7.0–5.0).

### 2.3 Probing pH‐ and Size‐Dependent Cargo Transmembrane Uptake of Avidin‐Psome B

Following the concept of Moreno et al.,^[^ ^75^ ^]^ FRET experiments (Figure S18, Supporting Information) were carried out to validate the pH‐dependent uptake of biotinylated horseradish peroxidase (Biotin‐HRP with *M*_w_ ≈ 44 kDa) and poly(ethylene glycol) (Biotin‐PEG_3kDa_) by collapsed, semi‐swollen, and swollen Avidin‐Psome B at pH 7.0, 6.0, and 5.0 in 1 × 10^−3^ m PBS.

The protocol for the preparation of HABA‐Avidin‐Psome B (HAAP B) to investigate the pH‐dependent uptake of cargo is shown in Figure 4a (very low level on starting fluorescence; see Figure S18, Supporting Information for details). Moreover, the pH* of Avidin‐Psome B is also not influenced by the presence of excess HABA (Figure S19b, Supporting Information). Then, Biotin‐HRP (1 eq) for HAAP B (HRP) sample or Biotin‐PEG_3kDa_ (2 eq) for HAAP B (PEG) sample were added to the HAAP B (HFF B1) solution at pH 7.0, 6.0, and 5.0, respectively. After stirring for 8 h, all samples were adjusted to pH 5.0 to immediately investigate the final fluorescence properties (*λ*_exc_ = 317 nm; *λ*_em_ = 518 nm) through the displacement of HABA in the binding pockets of avidin by Biotin‐HRP or Biotin‐PEG_3kDa_. To show the sequential cargo uptake (Figure 4a), HAAP B (HRP + PEG) sample was prepared by the addition of 1 eq Biotin‐PEG_3kDa_ to HAAP B (HRP) sample at various pH values.

For HAAP B (HRP) and HAAP B (PEG), the fluorescence intensity increases with decreasing pH values from 7.0 over 6.0 to 5.0 (Figure 4b; all fluorescent spectra in Figure S20, Supporting Information). Low fluorescence signals in all samples are observed in the collapsed state (pH 7.0), corroborating our previous hypothesis of a small amount of avidin anchored in the outer membrane or even weakly on the surface (Figure 3: location 4). However, fluorescent results for the displacement of HABA by Biotin‐PEG_3kDa_ at pH 5.0 for HAAP B (PEG) indicate the desired and complete membrane diffusion process of Biotin‐PEG_3kDa_ from outside to inside, visualized by the docking of Biotin‐PEG_3kDa_ to avidin biomacromolecules integrated in Avidin‐Psome B in the FRET experiment.

To further support a size‐dependent membrane diffusion of Biotin‐HRP in HAAP B (HRP), crossing HAAP B membrane from outside, additional experiments were carried out. An experiment at higher concentration (3 eq) of Biotin‐HRP for HAAP B (HRP) at pH 5.0 does not result in any increased fluorescence in the FRET experiment (Figure S20b, Supporting Information). Thus, the presence of excess Biotin‐HRP is not capable of overcoming the limited membrane diffusion of Biotin‐HRP in HAAP B (HRP).

However, the size‐ and pH dependent sequential uptake of Biotin‐HRP and Biotin‐PEG_3kDa_ by HAAP B membrane can be shown for HAAP B (HRP + PEG) (Figure 4). Indeed, the sequential addition of Biotin‐PEG_3kDa_ to HAAP B (HRP) further increases the fluorescence of HAAP B (HRP + PEG) in a pH‐dependent manner. Especially, the larger fluorescent increase by Biotin‐PEG_3kDa_ indicates the smooth membrane crossing of Biotin‐PEG_3kDa_ into the lumen of Avidin‐Psome B at pH 5.0. However, the fluorescent increase is much smaller at pH 6.0 and 7.0 compared to HAAP B (HRP), preferentially indicating only a slightly stronger docking process at the Psome B surface and first membrane integration at pH 7.0 and 6.0.

From these observations (Figure 4), a higher diffusion barrier is postulated for larger nanometer‐sized (macro)molecules to cross membrane of Avidin‐Psome B. In addition, once Biotin‐HRP enters in the swollen membrane at pH 5.0, an additional diffusion barrier can be fabricated by non‐covalent interactions between Biotin‐HRP and swollen membrane network and/or the formation of larger protein conjugates by HABA displacement in the swollen membrane. As a consequence, it is reasonable that HAAP B (HRP + PEG) results in a slightly lower fluorescence compared to HAAP B (PEG) (Figure 4b).

To conclude this part, it is possible to tailor in Avidin‐Psome B the location of biotinylated (macro)molecules in a pH‐ and size‐dependent manner, as highlighted in Figure 4a.

### 2.4 Probing the Enzymatic Metabolism of Avidin‐Psome B

As already verified, Biotin‐HRP can be docked on Avidin‐Psome B surface and within Avidin‐Psome B membrane, triggered by pH (Figure 4). In the following, Biotin‐HRP conjugated Avidin‐Psome B should act as artificial organelle to carry out simplified enzymatic metabolism. The protocol of this experiment series is presented in Figure 5a, where pure Avidin‐Psome B was fabricated and purified as mentioned above for AAF‐Psome B. We also used the same post‐loading concentration of Biotin‐HRP as presented in Figure 4 for the membrane diffusion experiments.

Biotin‐HRP solution was added to Avidin‐Psome B (HFF B1). Free Biotin‐HRP were removed by HFF purification. Finally, all Biotin‐HRP conjugated Avidin‐Psome B (HFF B1) samples were adjusted to pH 5.0, followed by HRP activity test. As control experiments (Figure S21, Supporting Information), Biotin‐HRP was also post‐loaded to Avidin‐Psome B (HFF B1‐3) (no avidin attached on the membrane) and empty Psome B, respectively. Thus, the HRP activity of Avidin‐Psome B (HFF B1‐3) is obviously higher than that of Avidin‐Psome B (HFF B1‐3) and empty Psome B samples (Figure 5b), which have similar HRP activity in the control experiment. Moreover, for all the samples with decreasing pH, the activity of HRP increases. This implies the increasing immobilization of Biotin‐HRP in a pH‐dependent manner and confirms the aforementioned postulation of Biotin‐HRP locations as indicated in HAAP B (HRP). Furthermore, the similar HRP activity of Avidin‐Psome B (HFF B1‐3) and empty Psome B further confirms the limited diffusion ability of Biotin‐HRP, which cannot diffuse through the membrane into the lumen of Psome B at pH 5.0.

### 2.5 Influence of Biotin‐PEG_3kDa_ Uptake on Conformational Changes of Psome B Determined by AF4‐LS

In order to investigate the conformational changes of Avidin‐Psome B after the uptake of Biotin‐PEG_3kDa_ at various pH, Avidin‐Psome B (HFF B1) was characterized before (as reference) and after the Biotin‐PEG_3kDa_ uptake using asymmetrical flow‐field flow fractionation with light scattering detection (AF4‐LS). The scaling plots of the samples with various uptake pH (5.0; 6.0, and 7.0) reveal a compact conformation of Avidin‐Psome B (HFF B1) with Biotin‐PEG_3kDa_ hybrids (Figure 6a). The scaling parameter *ν*  remains almost constant at pH 7, 6 and 5 with 0.41–0.35 indicating spherical shape, dense conformation with a slightly rough, irregular surface (Figure 6a) caused by avidin biomacromolecules on its surface. In theory, an ideal hard‐sphere has a parameter *ν* defined as 0.33.^[^ ^80^ ^]^

Moreover, the ratio of *R*_g_ and *R*_h_, (*ρ* parameter) confirms these assumptions (Figure 6b). For all samples, *ρ* values at concentration maximum around 1 are determined,^[^ ^81^ ^]^ characteristic for soft, spherical particles with a rough surface because of the presence of Biotin‐PEG_3kDa_ on the surface, except the sample at pH 5.0. At pH 5.0, *ρ* parameter is about 0.82, which is close to the definition of an ideal hard‐sphere.^[^ ^81^ ^]^ In addition, at pH 5.0 the taken‐up Biotin‐PEG_3kDa_ cargo is rather located inside (membrane and lumen) than at Psome B's surface compared to that at pH 6.0 and 7.0. These results are in accordance with the conclusion of FRET experiments that the biotinylated cargo uptake of Avidin‐Psome B is pH‐dependent (Figure 4).

The apparent densities further confirm our expectations that the pH value during the loading procedure of Avidin‐Psome B (HFF B1) has a significant influence on the incorporation efficiency of biotinylated PEG (Figure 6c). In addition, at pH 7.0, we detect no single surface‐modified Avidin‐Psome B (HFF B1), but particles with molar mass higher than 200 000 kg mol^−1^, showing that Avidin‐Psome B possesses a completely collapsed membrane. On the other hand, the Biotin‐PEG_3kDa_ cargo is not able to diffuse into the membrane or the lumen. Additionally to this, an interaction with the membrane surface is feasible, which preferably leads to further intermolecular assembly of Biotin‐PEG_3kDa_‐conjugated Avidin‐Psome B (HFF B1), resulting in higher density, large particles. The higher apparent density at pH 7.0 in comparison to the reference sample at high molar masses confirms this assumption. In contrast, at pH 6.0 (semi‐swollen state) and 5.0 (completely swollen state), the apparent densities are lower than the sample at pH 7.0. This is attributed to the radius increase of Avidin‐Psome B (HFF B1) after the successful uptake of Biotin‐PEG_3kDa_ at pH 6.0 (membrane) and 5.0 (membrane + lumen). Moreover, for the sample at pH 5.0, where the membrane is completely swollen, the Biotin‐PEG_3kDa_ uptake is much more effective. During the swelling process, a small reorganization of Psome B can be postulated, where a part of avidin–biotin hybrids diffuses into the inner part of Avidin‐Psome B (HFF B1), resulting in higher apparent density of the sample at pH 5.0 than that of the sample at pH 6.0.

### 2.6 Probing the Permeability Triggered by pH‐Dependent Cargo Diffusion in Coexisting Avidin‐Psome A and B Membranes

For mimicking the complexity of different organelles in real cells, our next step was to study the different pH‐dependent cargo diffusion process in coexisting Avidin‐Psome A and B (Figure 7).

For studying pH‐dependent orthogonal‐responsive membranes in the final experiment series (Figure 7), pH‐stable Avidin‐Psome A and B have been applied. Using the same approach as described for pH‐stable Avidin‐Psome B, abovementioned, pH‐stable Avidin‐Psome A is available showing no release of avidin at different pH values (Figures S16 and S17, Supporting Information).^[^ ^75^ ^]^ Moreover, we also used a sequential HFF purification method to collect information about avidin locations in Avidin‐Psome A (Figure S16, Supporting Information). Thus, compared to Avidin‐Psome B (Figure 3) less avidin is located in the lumen and inner membrane of Avidin‐Psome A (Figures S16 and S17, Supporting Information) attributed to the smaller membrane thickness.

Figure 7 (top) shows the different functional key characteristics (pH* and pH^0^) of pH‐responsive Psome A and B, determined by pH‐dependent DLS–titration in 1 × 10^−3^ m PBS buffer. Due to different pK_a_ values of DEAEMA (7.0–7.3)^[^ ^76^, ^77^ ^]^ in BCP‐A and of DPAEMA (≈6.0)^[^ ^78^, ^79^ ^]^ in BCP‐B (Figure 2) as well as other influencing factors,^[^ ^68^ ^]^ as already mentioned above, Psome A and B outline “open”, “closing/opening”, or “closed” states at different pH ranges (Figure 7, top). At pH range I (pH ≈ 4.0–4.9), Psome A and B are in “open” state, possessing large and uniform sizes. At pH range II (pH ≈ 4.9–6.0), Psome A is in “open” state. However, Psome B is in “closing/opening” state and their size decreases. At pH range III (pH ≈ 6.0–6.4), Psome A is in “open” state, and Psome B is in “closed” state. At pH range IV (pH ≈ 6.4–7.2), Psome A is in “closing/opening” state, and Psome B is in “closed” state. Finally, at pH range *V* (pH ≈ 7.2–8.0), Psome A and B are both in “closed” state. This undoubtedly shows the different protonation and deprotonation characteristics of Psome A and B as well as their different pH ranges at which both Psomes will steadily swell up to totally swollen state (= opening process).

Thus, various mixtures of Psome A and B were prepared to prove the sequentially pH‐driven membrane diffusion of cargo in the pH range between 5.0 and 7.0. To support this hypothesis, the cargo diffusion process in the presence of coexisting Psome A and B through FRET experiments was investigated. As shown in Figure 7 (bottom), the same concentration of AAF‐Psome A and B as well as empty Psome A and B solutions have been established for the final FRET experiments. Both AAF‐Psome A (HFF A1) (Figure S16, Supporting Information) and AAF‐Psome B (HFF B1) show similar fluorescent and quenching properties for preparing HAAP A (HFF A1) and HAAP B (HFF B1). The same concentration of Biotin‐PEG_3kDa_ and set up for cargo diffusion and fluorescent measurement were used as described for HAAP B (PEG) under FRET conditions (Figure 4).

Subsequently, Biotin‐PEG_3kDa_ was added to different HAAP A and B solutions at different pH values (Figure 7, bottom). Thus, Biotin‐PEG_3kDa_ can diffuse to different locations of Psome A or B in different “open” or “closed” state to displace HABA. Different increases of fluorescence intensity were expected to characterize the diffusion ability of Biotin‐PEG_3kDa_ through two different polymersome membranes at various pH values. To shortly exemplify this at pH 6.0, for Psome A, a complete membrane diffusion of Biotin‐PEG_3kDa_ from outside to inside is assumed besides outer membrane docking of Biotin‐PEG_3kDa_, while only first surface and outer membrane docking of Biotin‐PEG_3kDa_ for Psome B is hypothesized. Noteworthy, since the pockets occupied by the biotinylated compound cannot be quantified (maximum fluorescence is reached with 2 eq), this FRET experiment is a qualitative assay.^[^ ^75^ ^]^

The following samples in the presence of Biotin‐PEG_3kDa_ were investigated within this experiment series at pH 7.5, 7.0, 6.5, 6.0, 5.5, and 5.0: Mixture 1 consists of HAAP A (HFF A1) and empty Psome B; Mixture 2 unifies empty Psome A and HAAP B (HFF B1), Mixture 3 is composed of empty Psome A and HAAP B (HFF B1‐3), and Mixture 4 contains HAAP A (HFF A1) and HAAP B (HFF B1).

The resulting pH‐dependent fluorescence intensity of AAF‐Psome‐Biotin‐PEG_3kDa_ conjugates of all samples is shown in Figure 7 (bottom), while all fluorescence spectra are shown in Figure S23, Supporting Information. For all samples, different increasing fluorescent behavior from neutral to acidic is given. For Mixture 1, the HABA displacement indicates the expected closed (pH 7.5; docking on the surface), closing/opening (pH 7.0–6.0; preferred membrane diffusion), and open (pH 5.5 and 5.0; preferred diffusion into lumen) membrane for Psome A, with lower increase of fluorescence below pH 6.5. In opposite, Mixture 2 with HAAP B (HFF B1), outlines an extended pH period of closed membrane from pH 7.5 to pH 6.5 (same level on docking at outer surface) and, then, shows the expected opening membrane from pH 6.0 to pH 5.0 (membrane diffusion). At pH 5.0 for both Mixture 1 and 2, a similar high fluorescent intensity can be deduced. This implies that at this pH Biotin‐PEG_3kDa_ diffuses similarly into the lumen of Psome B and Psome A effectively. Compared with Mixture 2, Mixture 3 with HAAP B (HFF B1‐3) shows low fluorescence intensity at all studied pH values, indicating the already discussed lower remaining avidin loading, preferably in the membrane and lumen between pH 6 and 5. Finally, Mixture 4, loaded with HAAP A (HFF A1) and HAAP B (HFF B1), demonstrates the expected doubling of fluorescent intensity compared to Mixture 1 and 2, with a continuous increase of fluorescence intensity from pH 7.5 to 5.0, according to both opening pH ranges of the pH‐responsive polymersome membranes (Figure 7, bottom). Thus, this experiment series demonstrates for the first time the pH‐controlled cargo diffusion into two coexisting organelle mimics with different pH‐responsive behavior.

To conclude this part, the biotinylated cargo is able to dock on the outer surface and within the membrane of Psome A and B and to cross the opening and open membrane of Psome A and B, finally, diffusing into their lumen. The different intrinsic docking and diffusion processes of cargo (bio)macromolecules are pH‐dependent and in accordance with the pH titration DLS results of Psome A and B (Figure 7, top).

## 3 Conclusion

A new pH‐responsive and photo‐crosslinked Psome B was fabricated successfully as a candidate for artificial organelles, with a lower pH* (5.4, 1 × 10^−3^ m PBS) and a lower pH^0^ (6.0, 1 × 10^−3^ m PBS) compared to a previously reported analog Psome A (pH* 6.5 and pH^0^ 7.0).^[^ ^75^ ^]^ Avidin–Alexa Fluor 488‐Psome B (AAF‐Psome B) with the desired key characteristic of “non‐releasing avidin” was obtained after in‐situ loading and shear‐force driven HFF purification and by sequential dialysis at different pH values. Results of fluorescence spectroscopy after sequential HFF allowed to postulate that AAF‐488 conjugates possess a minority at location 4 (membrane outer surface) and a majority at location 3, 2, and 1 (in the membrane/inner membrane surface and lumen; Figure 3) in AAF‐Psome B. Avidin‐Psome B is able to mimic artificial organelles with uptake and docking of (bio)macromolecules in tailored locations in a pH‐ and size‐dependent manner. The transmembrane diffusion of biotinylated (bio)macromolecules increases with decreasing pH values from 7.0 over 6.0 to 5.0. This was also verified by the AF4 results after Biotin‐PEG_3kDa_ uptake at various pH values. As the cargo in a larger size, Biotin‐HRP cannot diffuse across the swollen membrane of Psome B at pH 5.0 and is only located at the outer and inner membrane. However, the smaller Biotin‐PEG_3kDa_ can diffuse fully through the swollen membrane into the lumen at pH 5.0, but not through the collapsed membrane at pH 6.0 and 7.0. Moreover, Biotin‐HRP conjugated Avidin‐Psome B can act as artificial organelles to enable simplified enzymatic metabolism. Finally, the permeability of artificial organelles triggered by the pH‐dependent cargo diffusion through coexisting Avidin‐Psome A and B membranes was probed. As a result, the biotinylated cargo, in this case Biotin‐PEG_3kD_, is able to dock on the outer surface and within the membrane of Psome A and B and to cross the opening and open membrane of Psome A and B, and finally diffuses into their lumen. The different intrinsic docking and diffusion processes of Biotin‐PEG_3kD_ as cargo are pH‐dependent and in accordance with the pH titration DLS results of Psome A and B. The cargo diffusion behavior through the membrane of coexisting Psome A and B mimics the cargo uptake process into different organelles, paving the way for the construction of multicompartmentalized protocell with the communications between different artificial organelles.

## 4 Experimental Section

##### Materials

The block copolymer (BCP‐A composition is presented in Table S1, Supporting Information; BCP‐B composition is presented in Figure 2a, Table S2, Supporting Information) used for polymersome fabrication to carry out in situ loading process of avidin and avidin–Alexa Flour 488 conjugates was synthesized by ATRP (further details of synthesis and characterization are in the Supporting Information). Avidin, avidin–Alexa Flour 488 conjugates, biotin, Biotin‐PEG_3kDa_ and Biotin‐HRP were purchased from Thermo Fisher. Other chemical information is shown in the Supporting Information.

##### Preparation of Polymersome A (Psome A) and Polymersome B (Psome B)

A solution of 1 mg mL^−1^ BCP‐A or BCP‐B in 0.01 m HCl (pH 2) was prepared and stirred overnight until the block copolymer was totally dissolved. The solution was passed through a nylon syringe filter (0.2 µm). Then, 1 m NaOH was added until pH was between 8.0 and 9.0 (Psome A) or between 5.6 and 6.0 (Psome B). The solution was stirred for 3 days. The final solution was passed through a cellulose ester syringe filter (0.8 µm) and placed in the UV chamber under irradiation for 90 s (Psome A) or 180 s (Psome B).

##### Reversible Swelling and Deswelling of Psome A and Psome B

1 m HCl or 1 m NaOH was added to 0.5 mg mL^−1^ Psome A or Psome B solution (0.01 m HCl solution) for obtaining pH 5.0 and pH 8.0 values (Psome A) or pH 4.0 and pH 7.0 values (Psome B), respectively. The diameter of Psome A or Psome B was determined by DLS and the test was repeated for 5 cycles.

##### pH* of Psome A and B Measurement

0.5 mg mL^−1^ Psome A or Psome B solution (≈10 × 10^−3^ m NaCl solution, 1 or 10 × 10^−3^ m PBS buffer, respectively) were titrated from basic to acidic conditions (pH values from 8.0 to 5.0 for Psome A, and from 7.0 to 4.0 for Psome B) while simultaneously measuring their size by DLS.

##### The Zeta Potential Values of Psome A and B at Different pH

1 m HCl or 1 m NaOH was added to 0.5 mg mL^−1^ Psome A or Psome B solution in 1 × 10^−3^ m PBS buffer for obtaining a set of samples at pH between 4.0 and 8.0. The ZP values of Psome A or Psome B in 1 × 10^−3^ m PBS buffer at different pH values were determined by Zetasizer Nano‐series instrument.

##### Psome A and B with Avidin–Alexa Flour 488 Conjugates by In‐Situ Loading

11 mg BCP‐A or B dissolved in 10 mL of 0.01 m HCl (pH 2) and stirred overnight until BCPs was totally dissolved. The solution was passed through a nylon syringe filter (0.2 µm). 0.1 m NaOH was added into 5.52 mL of filtered BCP‐A or B solution (1.1 mg mL^−1^) until pH was around 5.0. Then 0.48 mL of avidin–Alexa Flour 488 solution (1.25 mg mL^−1^) was added. Finally, to induce the self‐assembly process, 0.1 m NaOH was added increasing the pH to pH between 8.0 and 9.0 (BCP‐A) or between 5.6 and 6.0 (BCP‐B), respectively. After stirring for 3 days, the final solution was passed through a cellulose ester syringe filter (0.8 µm) and placed in the UV chamber under irradiation for 90 (BCP‐A) or 180 (BCP‐B) s, respectively.

It is worth noting that half amount of avidin–Alexa Flour 488 solution was added into Psome B solution to prepare AAF‐Psome B samples for FRET experiments.

##### HFF Purification of Polymersome Samples

HFF was carried out using KrosFlo Research Iii System equipped with a separation module made of polyethersulfone membrane (molecular‐weight cut‐off (MWCO): 500 kDa, SpectrumLabs, USA). The flow rate was 15 mL min^−1^ with the transmembrane pressure of 130 mbar. The shear‐forced separation of unbounded molecules was performed by washing the samples continuously with 1 × 10^−3^ m PBS buffer at pH 8.0 (Psome A) or pH 7.0 (Psome B) until no residues were observed in the waste solution.

##### Purification of AAF‐Psome A

6 mL of 1 mg mL^−1^ crude AAF‐Psome A solution was purified by HFF against 200 mL of 1 × 10^−3^ m PBS buffer at pH 8.0 called HFF A1. HFF purification of AAF‐Psome A after HFF A1 against 1 × 10^−3^ m PBS buffer at pH 7.0 is called HFF A2. HFF purification of AAF‐Psome A after HFF A2 against 1 × 10^−3^ m PBS buffer at pH 6.0 is called HFF A3.

##### Purification of AAF‐Psome B

6 mL of 1 mg mL^−1^ crude AAF‐Psome B solution was purified by HFF against 200 mL of 1 × 10^−3^ m PBS buffer at pH 7.0 called HFF B1. HFF purification of AAF‐Psome B after HFF B1 against 1 × 10^−3^ m PBS buffer at pH 6.0 is called HFF B2. HFF purification of AAF‐Psome B after HFF B2 against 1 × 10^−3^ m PBS buffer at pH 5.0 is called HFF B3.

The waste solution during the process of HFF and polymersome solution after HFF were collected and tested by fluorescence spectra (*λ*_excitation_ = 317 nm, *λ*_emission_ = 518 nm) at pH 5.0. After HFF the concentration of Psome A or Psome B was 0.66 mg mL^−1^.

##### Dialysis Purification of Polymersome B Samples

5 mL of 1 mg mL^−1^ crude Psome B with in‐situ loaded avidin–Alexa Flour 488 were transferred into the dialysis tubing (1000 kDa MWCO) and dialyzed against 1 × 10^−3^ m PBS buffer at pH 7.0 for 8, 24, and 48 h. After the dialysis, the sample was tested by fluorescence spectroscopy at the concentration of 0.66 mg mL^−1^ and at pH 5.0.

##### Sequential Dialysis Purification of Polymersome Samples after HFF

All the dialyses were carried out against 1 × 10^−3^ m PBS buffer in the dialysis tubing (1000 kDa MWCO).

##### Sequential Dialysis Purification of AAF‐Psome A

The procedure is shown in Figure S17, Supporting Information. The sequential dialysis of AAF‐Psome A (HFF A1) against 1 × 10^−3^ m PBS buffer for 8 h at pH 8.0, 7.0, and 6.0 are shown in Figure S17a, Supporting Information. Furthermore, the sequential dialysis of AAF‐Psome A (HFF A1‐2) against 1 × 10^−3^ m PBS buffer for 8 h at pH 7.0 and 6.0 (Figure S17b, Supporting Information), as well as the dialysis of AAF‐Psome A (HFF A1‐3) against 1 × 10^−3^ m PBS buffer for 8 h at pH 6.0 (Figure S17c, Supporting Information) was also carried out.

##### Sequential Dialysis Purification of AAF‐Psome B

The procedure is shown in Figure S14, Supporting Information. The sequential dialysis of AAF‐Psome B (HFF B1) against 1 × 10^−3^ m PBS buffer for 8 h at pH 7.0, 6.0 and 5.0 are shown in Figure S14a, Supporting Information. Furthermore, the sequential dialysis of AAF‐Psome B (HFF B1‐2) against 1 × 10^−3^ m PBS buffer for 8 h at pH 6.0 and 5.0 (Figure S14b, Supporting Information) as well as the dialysis of AAF‐Psome B (HFF B1‐3) against 1 × 10^−3^ m PBS buffer for 8 h at pH 5.0 (Figure S14c, Supporting Information) was carried out.

##### HABA Titration for AAF‐Psome Solution

8 mL of 0.66 mg mL^−1^ AAF‐Psome A (HFF A1) or AAF‐Psome B (HFF B1) in 1 × 10^−3^ m PBS buffer was adjusted to pH 5.0. Then ≈4–16 µL of 1 mg mL^−1^ HABA solution was added into 1 mL of 0.66 mg mL^−1^ AAF‐Psome A or B solution and tested by fluorescence spectroscopy.

##### pH‐ and Size‐Dependent Cargo Uptake to Psome B: HAAP B (HRP)

3 mL of HAAP B (HFF B1) solution was divided into three samples and adjusted to pH 7.0, 6.0 and 5.0, respectively. Afterwards, 10 µL of 2.5 mg mL^−1^ Biotin‐HRP solution was added to each sample at pH 7.0, 6.0, and 5.0, respectively. After stirring for 8 h, each sample was adjusted to pH 5.0 and tested by fluorescence spectroscopy.

##### pH‐ and Size‐Dependent Cargo Uptake to Psome B: HAAP B (HRP + PEG)

After checking the fluorescence spectroscopy, all the samples of HAAP B (HRP) were adjusted to pH 5.0. Afterwards, 10 µL of 2.5 mg mL^−1^ Biotin‐HRP solution was added to each sample at pH 7.0, 6.0, and 5.0, respectively. After stirring for 8 h, each sample was adjusted to pH 5.0 and tested by fluorescence spectroscopy. Then 11 µL of 0.1 mg mL^−1^ Biotin‐PEG_3kDa_ solution was added to each sample at pH 7.0, 6.0 and 5.0, respectively. After stirring for 8 h, each sample was adjusted to pH 5.0 and tested by fluorescence spectroscopy.

##### pH‐ and Size‐Dependent Cargo Uptake to Psome B: HAAP B (PEG)

3 mL of HAAP B (HFF B1) solution was divided into three samples and adjusted to pH 7.0, 6.0, and 5.0, respectively. Afterwards, 22 µL of 0.1 mg mL^−1^ Biotin‐PEG_3kDa_ solution was added to each sample at pH 7.0, 6.0 and 5.0, respectively. After stirring for 8 h, each sample was adjusted to pH 5.0 and tested by fluorescence spectroscopy.

##### Avidin‐Psome B Fabrication

11 mg BCP‐B dissolved in 10 mL of 0.01 m HCl (pH 2) and stirred overnight until BCPs was totally dissolved. The solution was passed through a nylon syringe filter (0.2 µm). 0.1 m NaOH was added into 5.52 mL of filtered BCP‐B solution (1.1 mg mL^−1^) until pH was around 5.0. Then 0.48 mL of avidin solution (0.625 mg mL^−1^) was added. Finally, to induce the self‐assembly process, 0.1 m NaOH was added increasing the pH to pH between 5.6 and 6.0, respectively. After stirring for 3 days, the final solution was passed through a cellulose ester syringe filter (0.8 µm) and placed in the UV chamber under irradiation for 180 s. Then the sample was divided into two samples and purified by HFF B1 and sequential HFF (HFF B1‐3, Figure 3), and after HFF, the concentration of all Avidin‐Psome B solution was 0.66 mg mL^−1^.

##### pH‐Dependent Biotin‐HRP Immobilization to Avidin‐Psome B

6 mL of 0.66 mg mL^−1^ Avidin‐Psome B (HFF B1) solution was divided into three samples and adjusted to pH 7.0, 6.0, and 5.0, respectively. After that, 20 µL of 2.5 mg mL^−1^ Biotin‐HRP solution was added to each sample. After stirring for 8 h, all the samples were purified by HFF with the flow rate of 15 mL min^−1^ and the transmembrane pressure of 70 mbar by washing the samples continuously with 40 mL of 1 × 10^−3^ m PBS buffer at pH 7.0. Afterwards, each sample at 0.66 mg mL^−1^ was adjusted to pH 5.0 and diluted by three‐fold. Then, 280 µL of diluted sample solution were taken out and mixed with 80 µL of 2 × 10^−3^ m ABTS solution, 40 µL of 35% w/w aqueous H_2_O_2_, followed by fluorescence spectrometer test at 414 nm from 0 s to 6 min.

As two parallel experiments, the same experiments were repeated on Avidin‐Psome B (HFF B1‐3) solution and empty Psome B in the same concentration, respectively.

##### pH‐Dependent Cargo Diffusion through Coexisting Avidin‐Psome A and B Membranes: Mixture 1

3.5 mL of 0.66 mg mL^−1^ HAAP A (HFF A1) was mixed with 3.5 mL of 0.66 mg mL^−1^ empty Psome B solution (after HFF B1) in 1 × 10^−3^ m PBS solution. Then the mixed polymersome solution was adjusted to pH 5.0 and 112 µL of 1 mg mL^−1^ HABA solution was added. After stirring for 2 h, 6 mL of mixed polymersome solution was divided into six samples and adjusted to pH 7.5, 7.0, 6.5, 6.0, 5.5, and 5.0, respectively. Then 22 µL of 0.1 mg mL^−1^ Biotin‐PEG_3kDa_ solution was added to each sample. After stirring for 8 h, all the samples were adjusted to pH 5.0 and tested by fluorescence spectroscopy.

##### pH‐Dependent Cargo Diffusion through Coexisting Avidin‐Psome A and B Membranes: Mixture 2

3.5 mL of 0.66 mg mL^−1^ HAAP B (HFF B1) was mixed with 3.5 mL of 0.66 mg mL^−1^ empty Psome A solution (after HFF A1) in 1 × 10^−3^ m PBS solution. Then the mixed polymersome solution was adjusted to pH 5.0 and 112 µL of 1 mg mL^−1^ HABA solution was added. After stirring for 2 h, 6 mL of mixed polymersome solution was divided into six samples and adjusted to pH 7.5, 7.0, 6.5, 6.0, 5.5, and 5.0, respectively. Then 22 µL of 0.1 mg mL^−1^ Biotin‐PEG_3kDa_ solution was added to each sample. After stirring for 8 h, all the samples were adjusted to pH 5.0 and tested by fluorescence spectroscopy.

##### pH‐Dependent Cargo Diffusion through Coexisting Avidin‐Psome A and B Membranes: Mixture 3

3.5 mL of 0.66 mg mL^−1^ HAAP B (HFF B1‐3) was mixed with 3.5 mL of 0.66 mg mL^−1^ empty Psome A solution (after HFF A1) in 1 × 10^−3^ m PBS solution. Then the mixed polymersome solution was adjusted to pH 5.0 and 112 µL of 1 mg mL^−1^ HABA solution was added. After stirring for 2 h, 6 mL of mixed polymersome solution was divided into six samples and adjusted to pH 7.5, 7.0, 6.5, 6.0, 5.5, and 5.0, respectively. Then 22 µL of 0.1 mg mL^−1^ Biotin‐PEG_3kDa_ solution was added to each sample. After stirring for 8 h, all the samples were adjusted to pH 5.0 and tested by fluorescence spectroscopy.

##### pH‐Dependent Cargo Diffusion through Coexisting Avidin‐Psome A and B Membranes: Mixture 4

3.5 mL of 0.66 mg mL^−1^ HAAP B (HFF B1) was mixed with 3.5 mL of 0.66 mg mL^−1^ HAAP A (HFF A1) in 1 × 10^−3^ m PBS solution. Then the mixed polymersome solution was adjusted to pH 5.0 and 112 µL of 1 mg mL^−1^ HABA solution was added. After stirring for 2 h, 6 mL of mixed polymersome solution was divided into six samples and adjusted to pH 7.5, 7.0, 6.5, 6.0, 5.5, and 5.0, respectively. Then 22 µL of 0.1 mg mL^−1^ Biotin‐PEG_3kDa_ solution was added to each sample. After stirring for 8 h, all the samples were adjusted to pH 5.0 and tested by fluorescence spectroscopy.

##### pH‐Dependent Cargo Diffusion through Coexisting Avidin‐Psome A and B Membranes: Mixture 5

3.5 mL of 0.66 mg mL^−1^ empty Psome A solution was mixed with 3.5 mL of 0.66 mg mL^−1^ empty Psome B solution in 1 × 10^−3^ m PBS solution. Then the mixed polymersome solution was adjusted to pH 5.0 and 112 µL of 1 mg mL^−1^ HABA solution was added. After stirring for 2 h, 6 mL of mixed polymersome solution was divided into six samples and adjusted to pH 7.5, 7.0, 6.5, 6.0, 5.5 and 5.0, respectively. Then 22 µL of 0.1 mg mL^−1^ Biotin‐PEG_3kDa_ solution was added to each sample. After stirring for 8 h, all the samples were adjusted to pH 5.0 and tested by fluorescence spectroscopy.

## Conflict of Interest

The authors declare no conflict of interest.

## Supporting information

Supporting Information

## Data Availability

Research data are not shared.
