# Supplementary material for: Artificial Organelles with Orthogonal‐Responsive Membranes for Protocell Systems: Probing the Intrinsic and Sequential Docking and Diffusion of Cargo into Two Coexisting Avidin–Polymersomes
Source: Adv Sci (Weinh). 2021 Apr 7;8(11):2004263. doi: 10.1002/advs.202004263 (PMC8188225; doi:10.1002/advs.202004263)
Supplement: Supplementary file 1 — Supporting Information [file ADVS-8-2004263-s001.pdf]

WILEY-VCH

© 2021 Wiley-VCH GmbH

ADVANCED SCIENCE
Open Access

Supporting Information

for *Adv. Sci.*, DOI: 10.1002/advs.202004263

Artificial Organelles with Orthogonal-Responsive Membranes for Protocells: Probing the Intrinsic and Sequential Docking and Diffusion of Cargo into Two Coexisting Avidin-Polymersomes

*Xueyi Wang, Silvia Moreno, Susanne Boye, Peng Wang, Xiaoling Liu, Albena Lederer, Brigitte Voit, Dietmar Appelhans**

Supporting Information

Artificial Organelles with Orthogonal-Responsive Membranes for Protocell Systems: Probing the Intrinsic and Sequential Docking and Diffusion of Cargo into Two Coexisting Avidin-Polymersomes

*Xueyi Wang, Silvia Moreno, Susanne Boye, Peng Wang, Xiaoling Liu, Albena Lederer, Brigitte Voit, Dietmar Appelhans**

*Corresponding author: applhans@ipfdd.de

Contents

1. Materials 6

2. Methods 6

NMR Spectroscopy. 6

Gel Permeation Chromatography (GPC) 6

Hollow Fiber Filtration (HFF). 7

Dynamic Light Scattering (DLS). 7

Zeta-potential measurements. 7

Fluorescence Spectroscopy. 7

UV lamp for crosslinking of polymersome. 7

UV-Vis Spectroscopy. 8

Cryogenic transmission electron microscopy (cryo-TEM). 8

Asymmetrical flow field flow fractionation (AF4). 8

3. Synthesis of block copolymers A and B 9

3.1. Synthesis of block copolymer A (BCP-A) 9

3.1.1. Synthesis of the PEG_{45}-Br macroinitiator (3) 9

3.1.2. Synthesis of the photo-crosslinker A (8) 10

3.1.3. Synthesis of the BCP-A (10) 10

3.2. Synthesis of block copolymer B (BCP-B) 11

3.2.1. Synthesis of the photo-crosslinker B (13) 11

3.2.2. Synthesis of the BCP-B (15) 12

4. pH-stability and avidin´s location of Avidin-Alexa Flour 488-Psome B (AAF-Psome B (HFF B1-2) and (HFF B1-3)) through approaches HFF 2 and 3 and fluorescence study 13

5. Short theoretical background for AF4-LS interpretation 14

6. Figures, Figure Captions and Tables 15

Table S1. The composition, molecular weight and dispersity (Đ) of BCP-A. [1] Determined by ^{1}H-NMR Spectroscopy, [2] Determined by GPC 15

Table S2. The composition, block ratio, crosslinker ratio and crosslinking time of different block copolymer B to fabricate stable Psome B. The composition, block ratio and crosslinker ratio are determined by ^{1}H NMR. 15

Table S3. Comparison of characteristics between Psome A and Psome B................................. 16

Table S4. Comparison of characteristics between AAF-Psome A and AAF-Psome B... 16

Table S5. Comparison of Biotin-PEG$_{3kDa}$ and Biotin-HRP.... 17

Table S6. Summary of AF4 measurements of empty and loaded Psome B samples (C_{BCP} = 0.5 mg/mL)... 18

Figure S1. Optimized separation flow profile of AF4 19

Figure S2. BCP-A synthesis using ATRP method. 19

Figure S3. ^{1}H NMR spectrum of BCP-A for determining the final structure. The corresponding atoms are marked in the polymer structure. 20

Figure S4. BCP-B synthesis using ATRP method.. 21

Figure S5. ^{1}H NMR spectrum of BCP-B for determining the final structure. The corresponding atoms are marked in the polymer structure. 22

Figure S6. The chemical structures of photo-crosslinker A and B, and the crosslinking reaction for dimer forming.. 22

Figure S7. The schematic figure for calculating starting point of swelling (pH^0), ending point of swelling (pH′) and turning point of pH-dependent size transition (pH*).. 23

Figure S8. DLS titration data of Psome A in 10 mM NaCl solution (a), 1 mM PBS buffer solution (b) and 10 mM PBS buffer solution (c) (• Diameter from DLS, ━ Logisitic fit of curve). (d) Zeta potential curves of Psome A in 1 mM PBS buffer at various pH values..................................... 24

Figure S9. (a) Reversible swelling/deswelling of Psome A in water at pH 5.0 and 8.0 by DLS. (b) Cryo-TEM micrograph of Psome A, scale bar 200nm. (Diameter Ø = 80.4 ± 17.2 nm, membrane thickness M = 16.1 ± 1.7 nm, measured from above 100 Psome A)... 24

Figure S10. DLS titration data of Psome B in 1 mM PBS buffer solution (a) and 10 mM PBS buffer solution (b) (• Diameter from DLS, ━ Logisitic fit of curve). (c) The pH* of Psome A and B in 10 mM NaCl solution, 1 mM and 10 mM PBS buffer solution, separately. (d) Zeta potential curves of Psome B in 1 mM PBS buffer at various pH values. 25

Figure S11. (a) The fluorescence spectra of avidin-Alexa Fluor 488-Psome B solution before and after purification by HFF 1 with 1 mM PBS buffer at pH 7.0 ($\lambda_{excitation}$ = 317 nm). (b) The

fluorescence intensity of free avidin-Alexa Fluor 488 in the waste solution during the process of HFF 1 with 1 mM PBS buffer at pH 7.0 ($\lambda_{excitation}$ = 317 nm, $\lambda_{emission}$ = 518 nm). 25

Figure S12. Protocol for the establishment of two-dimensional pH-stable avidin-Alexa Flour 488 loaded polymersome B (Avidin-Alexa Fluor 488-Psome B, AAF-Psome B) through sequential pH-dependent shear-force driven hollow-fiber-filtration (HFF). 26

Figure S13. The fluorescence spectra of Psome B with in-situ loaded avidin-Alexa Flour 488 before dialysis and after dialysis against 1 mM PBS buffer at pH 7.0 for 8 h, 24 h and 48 h, separately. 27

Figure S14. The protocol of purification of Avidin-Alexa Flour 488-Psome B by sequential dialysis (a, b, c) and the fluorescence spectra of AAF-Psome B (HFF B1), AAF-Psome B (HFF B1) a, AAF-Psome B (HFF B1) b and AAF-Psome B (HFF B1) c (d), AAF-Psome B (HFF B1-2), AAF-Psome B (HFF B1-2) a and AAF-Psome B (HFF B1-2) b (e), AAF-Psome B (HFF B1-3) and AAF-Psome B (HFF B1-3) a (f)... 28

Figure S15. (a) The fluorescence spectra of avidin-Alexa Fluor 488-Psome A solution before and after purification by HFF A1 with 1 mM PBS buffer at pH 8.0 ($\lambda_{excitation}$ = 317 nm). (b) The fluorescence intensity of free avidin-Alexa Fluor 488 in the waste solution during the process of HFF A1 with 1 mM PBS buffer at pH 8.0 ($\lambda_{excitation}$ = 317 nm, $\lambda_{emission}$ = 518 nm)........................ 29

Figure S16. Protocol for the establishment of pH-stable avidin-Alexa Flour 488 loaded Psome A (Avidin-Alexa Flour 488-Psome A, AAF-Psome A) through sequential pH-dependent HFF. Loading efficiency (%) of HFF purified Sample A1-A3 (right bottom) calculated from fluorescence spectra of Sample A1-A3 (left). pH-dependent DLS titration of Avidin-Psome A (right) for validating collapsed membrane, partially swollen membrane and highly swollen membrane...... 30

Figure S17. The protocol of purification of Avidin-Alexa Flour 488-Psome A by sequential dialysis (a, b, c) and the fluorescence spectra of Sample A1, A1a, A1b and A1c (d), Sample A2, A2a and A2b (e), Sample A3 and A3a (f). 31

Figure S18. Schematic representation of FRET experiment basis by HABA displacement. 32

Figure S19. (a) The fluorescence spectra of Avidin-Alexa Flour 488-Psome B (AAF-Psome B (HFF B1)) after adding different amount of HABA at pH 5.0. (b) DLS titration data of Avidin-Alexa Flour 488-Psome B (AAF-Psome B (HFF B1)) in 1 mM PBS buffer solution in presence of 16 µg/mL HABA. 32

Figure S20. The whole fluorescence spectra of samples in Figure 4. (a) The fluorescence spectra of HAAP B (HRP) at pH 5.0 after adding 25 µg/mL Biotin-HRP solution at different pH. (b) The fluorescence spectra of HAAP B (HRP) at pH 5.0 after adding 25 µg/mL and 75 µg/mL Biotin-

HRP solution at pH 5.0, respectively. (c) The fluorescence spectra of HAAP B (HRP + PEG) at pH 5.0 after adding 1.1 μg/mL 33

Figure S21. The protocol for enzyme assay of Biotin-HRP uptake to Avidin-Psome B after sequential HFF 1-3 (a) and empty Psome B (b) after adding Biotin-HRP and HFF purification. (The control experiment of Figure 5).. 34

Figure S22. (a) The fluorescence spectra of Avidin-Alexa Flour 488-Psome A (Sample A1) after adding different amount of HABA at pH 5.0. (b) DLS titration data of Avidin-Alexa Flour 488-Psome A (Sample A1) in 1 mM PBS buffer solution in presence of 16 μg/mL HABA. 35

Figure S23. The whole fluorescence spectra of samples in Figure 7 (bottom). (a) The fluorescence spectra of Mixture 1 (HABA-Avidin-Alexa Flour 488-Psome A and empty Psome B-HABA mixture) at pH 5.0 after adding 2.2 μg/mL Biotin-PEG3kDa solution at different pH. (b) The fluorescence spectra of Mixture 2 (HABA-Avidin-Alexa Flour 488-Psome B after HFF 1 and empty Psome A-HABA mixture) at pH 5.0 after adding 2.2 μg/mL Biotin-PEG3kDa solution at different pH. (c) The fluorescence spectra of Mixture 3 (HABA-Avidin-Alexa Flour 488-Psome B after sequential HFF 1-3 and empty Psome A-HABA mixture) at pH 5.0 after adding 2.2 μg/mL Biotin-PEG_{3kDa} solution at different pH. (d) The fluorescence spectra of Mixture 4 (HABA-Avidin-Alexa Flour 488-Psome A and HABA-Avidin-Alexa Flour 488-Psome B mixture) at pH 5.0 after adding 2.2 μg/mL Biotin-PEG3kDa solution at different pH.. 35

Figure S24. The fluorescence spectra of Mixture 5 (empty HABA-Psome A and B mixture) at pH 5.0 after adding 2.2 μg/mL 36

Figure S25. AF4 fractograms of different Psome B samples, LS and RI signals, and molar masses vs elution time. 37

Figure S26. (a) Scaling plots, R_g vs. molar masses and (b) apparent densities vs. molar masses of empty Psome B before and after HFF purification samples determined by AF4......................... 38

Figure S27. (a) Scaling plots, R_g vs. molar masses and (b) apparent densities vs. molar masses of Avidin-Psome B before and after HFF purification determined by AF4. 38

Figure S28. ρ parameter (R_g/R_h) vs molar masses of Avidin-Psome B, HFF purified with different Biotin-PEG_{3kDa} loading conditions (pH 5.0…red; pH 6.0…green; pH 7.0…blue) determined by AF4.. 39

7. References 39

1. Materials

All chemicals were used without any further purification, unless otherwise stated. Copper (I) bromide, 2-bromoisobutyryl bromide, 2,2′-bipyridine, aluminum oxide (neutral, activated), Brockmann I grade, 2,3-Dimethylmaleic anhydride, poly(ethylene glycol) methyl ether (MeO-PEG_{45}-OH, Mn: 2000 g/mol, ĐM:1.05), 2-(diethylamino)ethyl methacrylate (DEAEMA), 2-(Diisopropylamino)ethyl methacrylate (DPAEMA), tetrahydrofuran, 2-butanone, triethylamine, phosphate buffered saline tablets (PBS), sodium hydroxide (NaOH), 4-Amino-1-butanol, methacryloylic chloride, 2-(4-Hydroxyphenylazo)benzoic acid (HABA) were purchased from Sigma-Aldrich. 6-Amino-1-hexanol were purchased from TCI. Avidin, avidin-Alexa Fluor 488 conjugate, biotin, invitrogen™ biotinylated peroxidase (Biotin-HRP) and Biotin-PEG_{3kDa} were purchased from Thermo Fisher. Ethyl acetate and n-hexane were purchased from Fisher Chemical. Anhydrous toluene was purchased from Acros Organics and stored with molecular sieves. Dialysis membranes made of cellulose ester (molecular weight cut-off 1 kDa and 1000 kDa), 0.2 µm nylon filter and 0.8 µm cellulose mixed ester (CME) filter were purchased from Carl Roth.

2. Methods

NMR Spectroscopy. Bruker Avance III 500 spectrometer (Bruker Biospin, Germany) is used as the device for recording ^1H NMR (500.13 MHz) spectra with $CDCl_3$, DMSO-d_6 as a solvent at room temperature. Chemical shifts were expressed in ppm and referenced to corresponding solvent signals ($CDCl_3$: δ=7.26 ppm; DMSO-d_6: δ=2.50 ppm).

Gel Permeation Chromatography (GPC). The weight average molecular weight (M_w), number average molecular weight (M_n) and molar mass distributions (Đ) of block copolymers were measured using SEC equipped with a MALLS detector (MiniDAWN-LS detector, Wyatt

Technology, USA) and a viscosity/refractive index (RI) detector (ETA-2020, WGE Dr. Bures, Germany). The column (PL MIXED-C with a pore size of 5 µm, 300x7.5 mm) and the pump (HPLC pump, Agilent 1200 series) were from Agilent Technologies (USA). THF was used as an eluent (stabilized with 0.025 % BHT) with a flow rate of 1 mL/min. The calibration was based on polystyrene standards ranging from 1300 to 377400 g/mol.

Hollow Fiber Filtration (HFF). HFF was carried out using KrosFlo Research Iii System. This device was equipped with a separation module made of polyether sulfone membrane (MWCO: 500 kDa, SpectrumLabs, USA). The transmembrane pressure was from 70 mbar to 150 mbar with a flow rate of 15 mL/min. All the samples were purified by washing continuously with 1 mM PBS buffer at pH from 8.0 to 5.0.

Dynamic Light Scattering (DLS). DLS measurements of aqueous polymersome solutions (0.2 ~ 1 mg/mL) were performed using a Zetasizer Nano-series instrument (Malvern Instruments, UK) equipped with Dispersion Technology Software (version 5.00). The measurements were carried over a range of pH at 25°C. The data was collected using the NIBS (non-invasive back-scatter) method using a Helium–Neon laser (4 mW, l = 632.8 nm) and a fixed angle of 173°.

Zeta-potential measurements. Zeta potential of the polymersome solution (0.5 mg/mL) was determined by Zetasizer Nano-series instrument (Malvern Instruments, UK) through electrophoretic light scattering.

Fluorescence Spectroscopy. Fluorescence spectra were measured Fluorolog 3 (Horiba JobinYvon, USA) fluorescence spectrophotometer at 25°C.

UV lamp for crosslinking of polymersome. EXFO Omnicure 1000 (Lumen Dynamics GroupInc., Canada) equipped with a high-pressure mercury lamp as UV source was used for

crosslinking the polymersomes. The crosslinking process were performed with 1~1.5 mL of polymersome solution for from 90 to 180 seconds.

UV-Vis Spectroscopy. UV-vis analysis was performed using Specord 210 Plus double beam UV-vis spectrophotometer (analytikjena, Germany). Samples were measured at desired wavelength range in semi-micro cuvettes (Brand GmbH).

Cryogenic transmission electron microscopy (cryo-TEM). Cryo-TEM images were acquired using Libra 120 microscope (Carl Zeiss Microscopy GmbH, Oberkochen, Germany) at an acceleration voltage of 120 kV. Samples were prepared by dropping 2 μL of polymersome solution on copper grids coated with holey carbon foil (so-called Lacey type). A piece of filter paper was used to remove the excess water; the sample was then rapidly frozen in liquid ethane at -178 °C. The blotting with the filter paper and plunging into liquid ethane was done in a Leica GP device (Leica Microsystems GmbH, Wetzlar, Germany). All images were recorded in bright field at -172 °C. The diameter and membrane thickness of the polymersome were determined from cryo-TEM images by using ImageJ Sofware. The average of polymersome diameter was calculated by analyzing more than 100 particles. The average of membrane thickness was calculated by analyzing 30 particles.

Asymmetrical flow field flow fractionation (AF4). AF4 measurements were performed with an Eclipse DUALTEC system (Wyatt Technology Europe, Germany) with 1 mM PBS buffer at pH 6.5, containing 200 mg/L NaN_3 to prevent bacteria or algae contamination as carrier liquid. The channel spacer, made of poly(tetrafluoroethylene), had a thickness of 490 μm. The dimensions of the channel were 26.5 cm in length and from 2.1 to 0.6 cm in width. The membranes used as accumulation wall were composed of polyethersulfone with a molecular weight cut off (MWCO) of 10 kDa (Nadir, Germany). Flow rates were controlled with an Agilent Technologies 1260er series isocratic pump equipped with vacuum degasser. The

detection system consisted of a MALS detector (DAWN HELEOS II, Wyatt Technology Europe, Germany) operating at a wavelength of 659 nm with online DLS detector (QELS module, Wyatt Technologies, USA) which is an add-on unit connected to the 99° angle of the MALS, a diode array detector SPD-M20A (Shimadzu Europe) and a refractive index (RI) detector (Optilab T-rEX, Wyatt Technology Europe GmbH, Germany) operating at a wavelength of 658 nm. All injections were performed with an autosampler (1260 series, Agilent Technologies Deutschland GmbH). The data collection and calculation of molecular weights and radii were performed by Astra 7.3.219 software (Wyatt Technologies, USA). Cross flow rate (Fx) profile was optimized to achieve optimal fractionation of free molecules from Psomes within the same elution (Figure S1). The following protocol was applied: detector flow was set to 0.5 mL/min, focusing was performed with focus flow (Ff) 2.5 mL/min for 4 min followed by an isocratic elution step with a Fx of 2 mL/min for 5 min followed by an exponential Fx gradient from 2 to 0 mL/min within 20 min. The last step proceeds without Fx (0 mL/min) for 15 min. Three injections of 200 µL were performed for each sample. Mw and radius of gyration (R_g) of Psomes were calculated from the MALS data of detectors 6 to 17 applying a Berry fit.

3. Synthesis of block copolymers A and B

3.1. Synthesis of block copolymer A (BCP-A)

Block copolymer A (BCP-A) was synthesized according to our previous published approach[1, 2] showed in Figure S2.

3.1.1. Synthesis of the PEG_{45}-Br macroinitiator (3)

The PEG_{45}-Br macroinitiator was synthesized according to our previous published approach[1, 2]. Here, MeO-PEG_{45}-OH (5.00 g, 2.5 mmol) (1) was dissolved in THF (45 mL). Then triethylamine (0.74 g 4 mmol) and 2-bromoisobutyric acid bromide (1.12 g, 4.9 mmol) (2) dissolved in THF (3 mL) and were added into MeO-PEG_{45}-OH solution under nitrogen

atmosphere. The reaction was carried out for 3 days at room temperature under nitrogen atmosphere. The raw product was precipitated in cooled ether and died in vacuum oven. Finally, white solid was obtained as the macroinitiator. Yield: 48 %.

^{1}H NMR (500 MHz, $CDCl_3$) δ 4.36 (s, 2H), 3.67 (s, 180H), 3.41 (s, 3H), 1.97 (s, 6H).

3.1.2. Synthesis of the photo-crosslinker A (8)

Photo-crosslinker A (8) was synthesized in two steps.

Synthesis for step 1: Aminobutanol (1.2 g, 14 mmol) (5) was dissolved in 100 mL anhydrous toluene, then maleic acid anhydride (1.71 g, 14 mmol) (4) was added. The mixture was kept at reflux for 2 h at a water trap. The raw product was obtained after rotary evaporation, and finally purified by flash chromatography (n-hexane/ethyl acetate, 50:50 vol-%) to get the compound 6. Yields: 79 %.

^{1}H NMR (500 MHz, $CDCl_3$) δ 3.58 (t, 2H), 3.46 (t, 2H), 1.95 (s, 6H), 1.66 (quin, 2H), 1.51-1.57 (m, 3H).

Synthesis for step 2: Compound 6 (2 g, 10.1 mmol) was dissolved in anhydrous THF (117 mL) and then cooled in ice-water. After cooling, methacryloyl chloride (1.63 g, 15.6 mmol) (7) dissolved in THF (3 mL) was added. The reaction proceeded for overnight at room temperature. After reaction, the mixture was extracted with diethyl ether, then dried over anhydrous magnesium sulfate and removed by rotary evaporation sequentially to obtain the raw product of photo-crosslinker A (8), which was finally purified by flash chromatography (n-hexane/ethyl acetate, 75:25 Vol-%). Yields: 56%.

^{1}H NMR (500 MHz, $CDCl_3$) δ 6.09 (s, 1H), 5.55 (s, 1H), 4.15 (t, 2H), 3.53 (t, 2H), 1.94 (s, 3H), 1.67-1.69 (m, 4H).

3.1.3. Synthesis of the BCP-A (10)

BCP-A was synthesized according to our previous published approach by ATRP[1, 2].

PEG_{45}-Br macroinitiator ((107 mg, 0.05 mmol) (3), 2,2′-bipyridine (14.5 mg, 0.093 mmol), DEAEMA (604 mg, 3.257 mmol) (9), and photo-crosslinker A (247 mg, 0.931 mmol) (8) were mixed in a Schlenk tube with a stirring bar. The compounds were dissolved in 2-butanone (1.5 mL) and completely frozen by liquid nitrogen. Then CuBr (6.7 mg, 0.047 mmol) was added into the Schlenk tube, and the mixture is degassed using three freeze-pump-thaw-cycles, backfilled with argon and stirred overnight at 50°C. To end the polymerization the tube is cooled and opened, the reaction solution was diluted with THF and filtrated over aluminium oxide to remove all copper species. The reaction solution was concentrated by rotary evaporation and then precipitated in cooled n-hexane to obtain BCP-A (10) after vacuum drying. Yield: 83 %.

The ^{1}H NMR result of BCP-A is showed in Figure S3.

3.2. Synthesis of block copolymer B (BCP-B)

All the reaction schemes for the preparation of block copolymers B (BCP-B) are showed in Figure S4.

3.2.1. Synthesis of the photo-crosslinker B (13)

Photo-crosslinker B (13) was synthesized according to our previous published approach[1, 2].

Synthesis for step 1: 6-Amino-1-hexanol (1.6 g, 14 mmol) (11) was dissolved in 100 mL anhydrous toluene, then maleic acid anhydride (1.71 g, 14 mmol) (4) was added. The mixture was kept at reflux for 2 h at a water trap. The raw product was obtained after rotary evaporation, and finally purified by flash chromatography (n-hexane/ethyl acetate, 50:50 vol-%) to get compound 12. Yields: 75 %.

^{1}H NMR (500 MHz, $CDCl_3$) δ 3.63 (t, 2H), 3.49 (t, 2H), 1.96 (s, 6H), 1.58 (quin, 4H), 1.46 (s, 1H), 1.40 (m, 2H), 1.33 (m, 2H).

Synthesis for step 2: Compound 12 (2.2 g, 10.1 mmol) was dissolved in anhydrous THF (117 mL) and then cooled in ice-water. After cooling, methacryloyl chloride (1.63 g, 15.6 mmol) (7) dissolved in THF (3 mL) was added. The reaction proceeded for overnight at room temperature. After reaction, the mixture was extracted with diethyl ether, then dried over anhydrous magnesium sulfate and removed by rotary evaporation sequentially to obtain the raw product of photo-crosslinker B (8), which was finally purified by flash chromatography (n-hexane/ethyl acetate, 75:25 Vol-%). Yields: 51%.

^{1}H NMR (500 MHz, $CDCl_3$) δ 6.09 (s, 1H), 5.54 (s, 1H), 4.12 (t, 2H), 3.47 (t, 2H), 1.98 (s, 6H), 1.96 (s, 3H), 1.65-1.58 (m, 4H), 1.39-1.31 (m, 4H).

3.2.2. Synthesis of the BCP-B (15)

BCP-B was synthesized according to our previous published approach by ATRP[1, 2].

PEG_{45}-Br macroinitiator ((107 mg, 0.05 mmol) (3), 2,2′-bipyridine (14.5 mg, 0.093 mmol), DPAEMA (666 mg, 3.35 mmol) (14), and photo-crosslinker B (314.1 mg, 1.15 mmol) (13) were mixed in a Schlenk tube with a stirring bar. The compounds were dissolved in 2-butanone (1.5 mL) and completely frozen by liquid nitrogen. Then CuBr (6.7 mg, 0.047 mmol) was added into the Schlenk tube, and the mixture is degassed using three freeze-pump-thaw-cycles, backfilled with argon and stirred overnight at 50°C. To end the polymerization the tube is cooled and opened, the reaction solution was diluted with THF and filtrated over aluminium oxide to remove all copper species. The reaction solution was concentrated by rotary evaporation and then precipitated in cooled n-hexane to obtain BCP-B (15) after vacuum drying. Yield: 87 %

The ^{1}H NMR result of BCP-B is showed in Figure S5.

4. pH-stability and avidin´s location of Avidin-Alexa Flour 488-Psome B (AAF-Psome B (HFF B1-2) and (HFF B1-3)) through approaches HFF 2 and 3 and fluorescence study

The fluorescence intensity of AAF-Psome B (HFF B1-2) after purification HFF B1-2 decreases from around 2.8×10^6 (AAF-Psome B (HFF B1)) to around 1.9×10^6. This further results in a reduction of loading efficiency (17.3 ± 1.5%) for avidin-Alexa Flour 488 conjugates triggered by the shear-force driven HFF 2 elimination of avidin-Alexa Flour 488 conjugates at pH 6.0 in 1 mM PBS (Figure 3) mainly at the polymerome surface and a lower content of membrane-integrated avidin-Alexa Flour 488 conjugates. This assumption is supported by the presence of still collapsed Psome B membrane at pH 6.0 which provides the starting point of swelling (pH^0) to lower pH values (Figure 3). Thus, we assume that the residual avidin-Alexa Flour 488 conjugates in AAF-Psome B (HFF B1-2) (Figure 3) is located at inner hydrophilic shell of membrane (location 2), hydrophobic membrane (location 3) and lumen (location 1).

The fluorescence intensity of AAF-Psome B (HFF B1-3) decreases only from around 1.9×10^6 (AAF-Psome B (HFF B1-2)) to around 1.7×10^6. A low reduction (< 2%) of loading efficiency for avidin-Alexa Flour 488 conjugates (15.5 ± 1.8%) is obtained. Obviously, only a tiny part of avidin-Alexa Flour 488 conjugates is released from Psome B during HFF 3 purification (Figure 3). We hypothesize that the avidin-Alexa Flour 488 conjugates is mainly released from hydrophobic membrane (location 3) at highly swollen membrane and that after 3-times applied HFF the most avidin-Alexa Flour 488 conjugates is located at the lumen and inner hydrophilic shell of membrane and less of avidin-Alexa Flour 488 conjugates in the hydrophobic membrane (Figure 3).

Furthermore, the sequential dialysis of AAF-Psome B (HFF B1-2) against 1 mM PBS buffer for 8 h at pH 6.0 and 5.0 (Figure S14b, e) as well as the dialysis of AAF-Psome B (HFF B1-3) against 1 mM PBS buffer for 8 h at pH 5.0 (Figure S14c, f) was also carried out. The results imply that there is a negligible release of avidin-Alexa Flour 488 conjugates from AAF-Psome B (HFF B1-2) and AAF-Psome B (HFF B1-3) over dialysis at low pH, furtherly confirming the stability of Avidin-Psome B without avidin release.

5. Short theoretical background for AF4-LS interpretation

Scaling parameter: by plotting R_g vs M, ν can be determined by the slope of the plot, it gives information about the molecular shape in the used solvent.

$R_g = K \cdot M^{\nu}$

ν = 0.33 → spheres

ν = 0.5 - 0.6 → random coil macromolecule

ν = 1 → rigid rod

Apparent density: gives information about molecular density, is calculated by R_g and M_w (with V as volume fraction, α as geometrical correction, N_A as Avodgadro's number):

$$d_{app,i} = \frac{M_i}{V\,(R_g)_i \cdot N_A} \cdot \alpha \qquad \text{with} \qquad \alpha = \frac{V_{sphere}(R_g)}{V_{sphere}(R)} = \frac{R_g{}^3}{R^3} = \frac{\left(\sqrt{\frac{3}{5}} \cdot R\right)^3}{R^3} = \left(\frac{3}{5}\right)^{\frac{3}{2}}$$

ρ parameter: The ratio between R_g and R_h delivers valuable information about conformation and shape of molecules, some examples[3]:

Homogenous sphere:	0.775
Random coil, linear chain (good solvent):	1.78
Hyperbranched polymer:	1.23
Rod (axial ratio = 2.5):	2.1

6. Figures, Figure Captions and Tables

Table S1. The composition, molecular weight and dispersity (Đ) of BCP-A. [1] Determined by ^{1}H-NMR Spectroscopy, [2] Determined by GPC.

Polymer	Monomer amount[1]	Crosslinker amount[1]	PEG amount[1]	M_w (Kg/mol)[1]	M_w (Kg/mol)[2]	M_n (Kg/mol)[2]	Đ[2]
BCP-A	70	20	45	20.5	33.6	28.6	1.18

Table S2. The composition, block ratio, crosslinker ratio and crosslinking time of different block copolymer B to fabricate stable Psome B. The composition, block ratio and crosslinker ratio are determined by ^{1}H NMR.

Name	Monomer amount[1]	Crosslinker amount[1]	PEG amount[1]	Block ratio[1]	Crosslinker ratio[1]	Crosslinking time (s)	Stable Psome B
BCP B1	71	23	45	1:2.08	24.5% (A)	90	-
BCP B1	71	23	45	1:2.08	24.5% (A)	180	-
BCP B2	73	23	45	1:2.13	23.9% (B)	90	-
BCP B2	73	23	45	1:2.13	23.9% (B)	180	-
BCP B3	59	28	45	1:1.93	32.2% (A)	90	-
BCP B3	59	28	45	1:1.93	32.2% (A)	180	-
BCP B4	57	27	45	1:1.86	32.1% (B)	90	-
BCP B4	57	27	45	1:1.86	32.1% (B)	140	-
BCP B4	57	27	45	1:1.86	32.1% (B)	180	√

- means polymersomes are not stable under various pH

√ means polymersomes are stable under various pH

Table S3. Comparison of characteristics between Psome A and Psome B.

Psome	Diameter (nm)	Membrane thickness (nm)	pH* (1 mM PBS)	pH^0 (1 mM PBS)	pH´ (1 mM PBS)
Psome A	80.4 ± 17.2	16.1 ± 1.7	6.8	7.2	6.4
Psome B	120.6 ± 32.3	26.9 ± 2.9	5.4	6.0	4.8

pH* means turning point of pH-dependent size transition

pH^0 means starting point of pH-dependent size transition

pH´ means ending point of pH-dependent size transition

Table S4. Comparison of characteristics between AAF-Psome A and AAF-Psome B.

Psome	pH* (1 mM PBS)	Avidin loading efficiency (after HFF 1)	Avidin loading efficiency (after HFF 1-2)	Avidin loading efficiency (after HFF 1-3)	Avidin releasing from AAF-Psome
AAF-Psome A	6.8	10.3 ± 0.4%	7.9 ± 0.8%	6.7 ± 0.5%	Non
AAF-Psome B	5.4	25.9 ± 2.2%	17.3 ± 1.5%	15.5 ± 1.8%	Non

Table S5. Comparison of Biotin-PEG_{3kDa} and Biotin-HRP.

Cargo	Molecular Weight (kDa)	Shape	Zeta potential (mV)
Biotin-PEG_{3kDa}	3.2	Linear	-1.25
Biotin-HRP	44	Spherical	-0.13

A short explanation: The differences between Biotin-PEG_{3kDa} and Biotin-HRP are the molecular weight (Biotin-PEG_{3kDa}: 3.2 kDa; Biotin-HRP: 44 kDa) and shape (Biotin-PEG_{3kDa}: linear; Biotin-HRP: spherical). The surface charge under the studied conditions is similar in both cases (zeta potential (Biotin-PEG_{3kDa}: -1.25; Biotin-HRP: -0.13)). Biotin-HRP has a much higher molecular weight. According to our previously published paper,[4] molecular weight is the key factor for the cargo uptake into pH-responsive polymersome. No significant differences between linear and spherical cargo in the uptake can be concluded from recent and previous results.[4] The effect of surface charge is slight, but it is difficult to generalize due to retarding properties of cationic avidin biomacromolecules in protonated polymersome membrane in recent and previous results.[4] Thus, the results of sequential uptake of Biotin-PEG_{3kDa} and Biotin-HRP indicate the uptake in molecular weight-dependent manner, not shape- or surface charge-dependent manner.

Table S6. Summary of AF4 measurements of empty and loaded Psome B samples (C_{BCP} = 0.5 mg/mL).

Psome sample	M_n*1 (kg/mol)	M_w*1 (kg/mol)	Đ (M_w/M_n)	R_g (nm)	R_h (nm)	ρ (R_g/R_h)*2	Apparent Density (kg/m³)
**Empty Psome B,** unpurified	5.07 e5	6.84 e5	1.17	92.0	95.5	1.06	161.8
**Empty Psome B,** HFF purified	5.76 e5	8.64 e5	1.50	97.6	76.1	1.32	171.2
**Avidin-Psome B,** physical mixture, **unpurified**	2.51 e5	7.33 e5	3.09	108	69.4	1.35	107.2
**Avidin-Psome B**, *in-situ*, **unpurified**	2.93 e4	2.11 e5	7.43	102	47.5	1.33	36.6
**Avidin-Psome B, *in-situ*, HFF purified**	1.45 e5	2.40 e5	1.69	70.8	71.0	1.00	124.6
**Biotin-PEG_{3kDa}+Avidin-Psome B, *post* at pH 5.0**	9.29 e4	2.19 e5	2.38	92.2	136	0.80	51.5
Biotin-**PEG_{3kDa}+Avidin-Psome B, *post* at pH 6.0**	1.82 e5	4.44 e5	2.44	104	108	0.94	29.8
Biotin-**PEG_{3kDa}+Avidin-Psome B, *post* at pH 7.0**	1.13 e6	1.56 e6	1.39	123	132	1.04	154.0

*1…calculated with dn/dc = 0.183 mL/g

*2…peak maximum of concentration signa

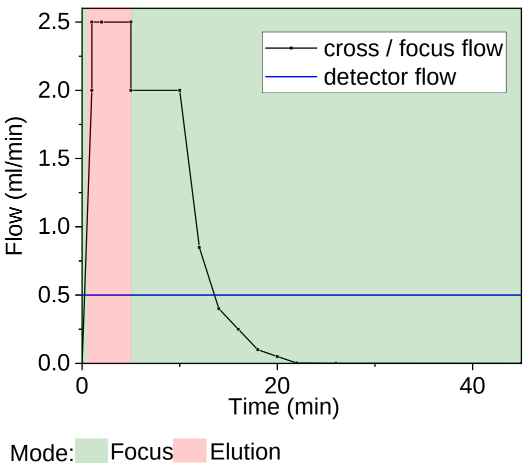

Figure S1. Optimized separation flow profile of AF4.

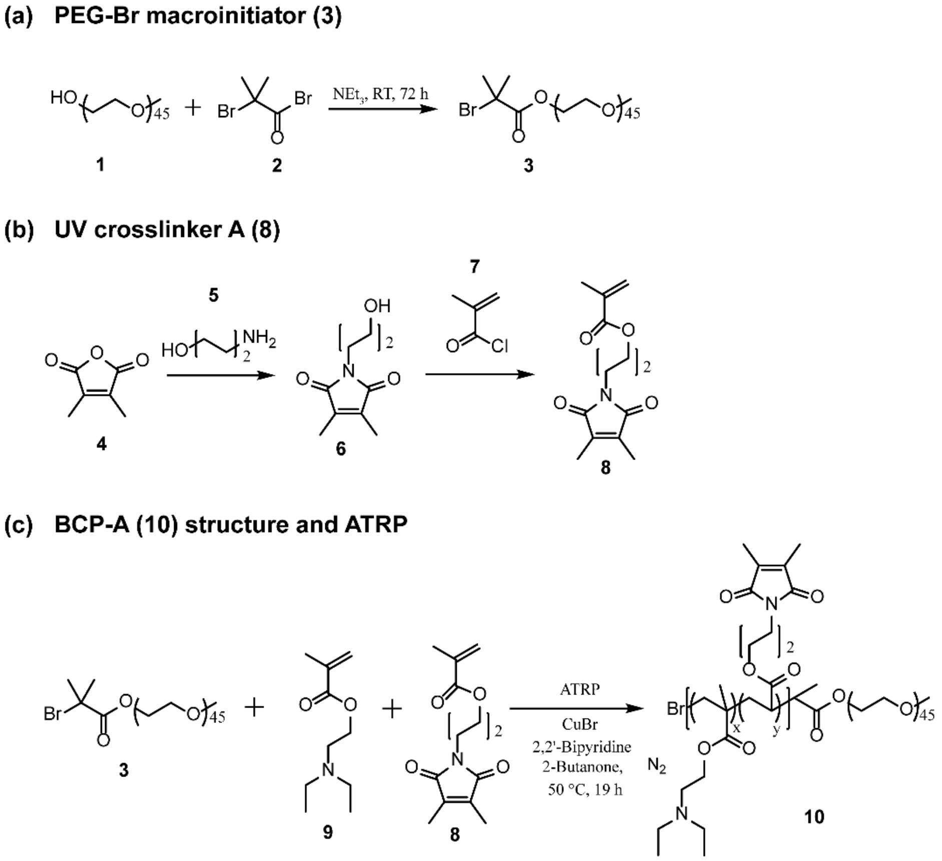

Figure S2. BCP-A synthesis using ATRP method**.**

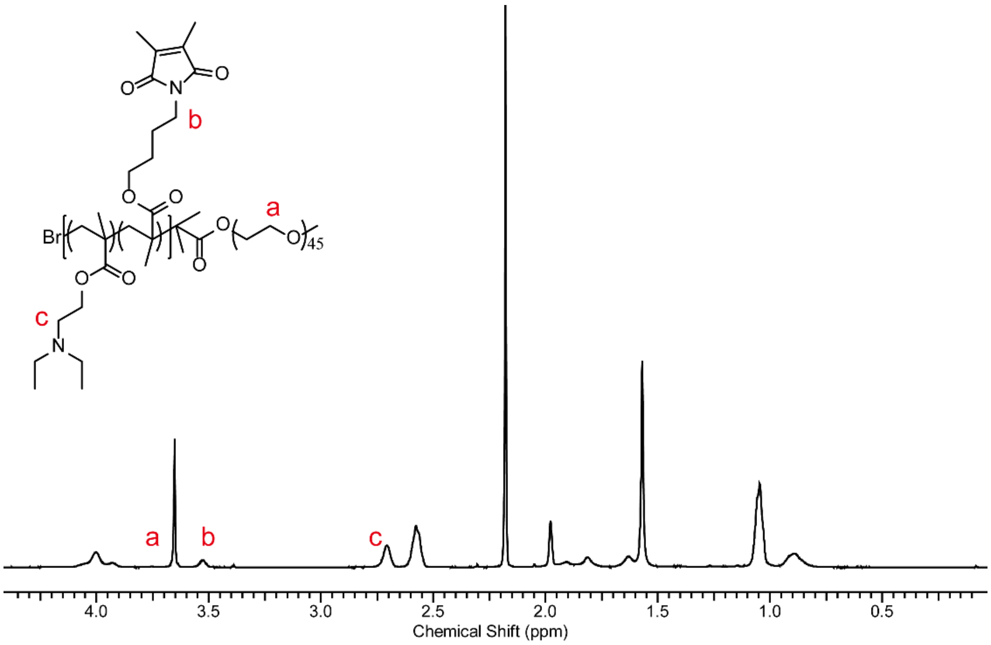

Figure S3. ^{1}H NMR spectrum of BCP-A for determining the final structure. The corresponding atoms are marked in the polymer structure.

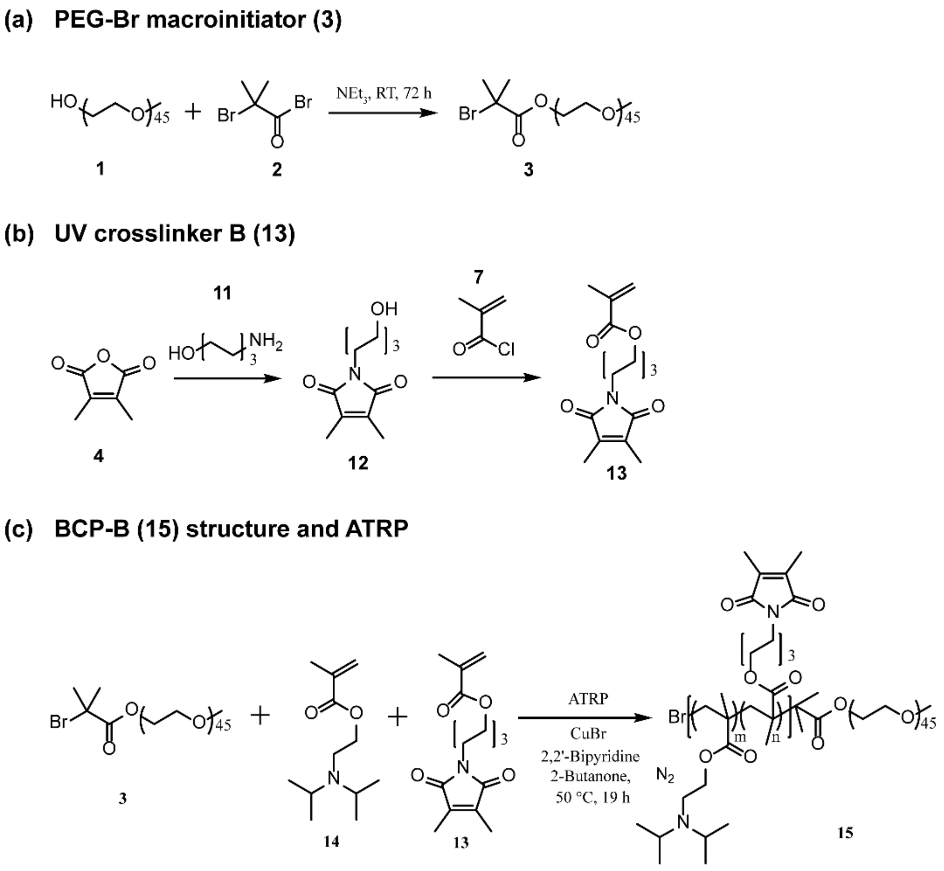

Figure S4. BCP-B synthesis using ATRP method.

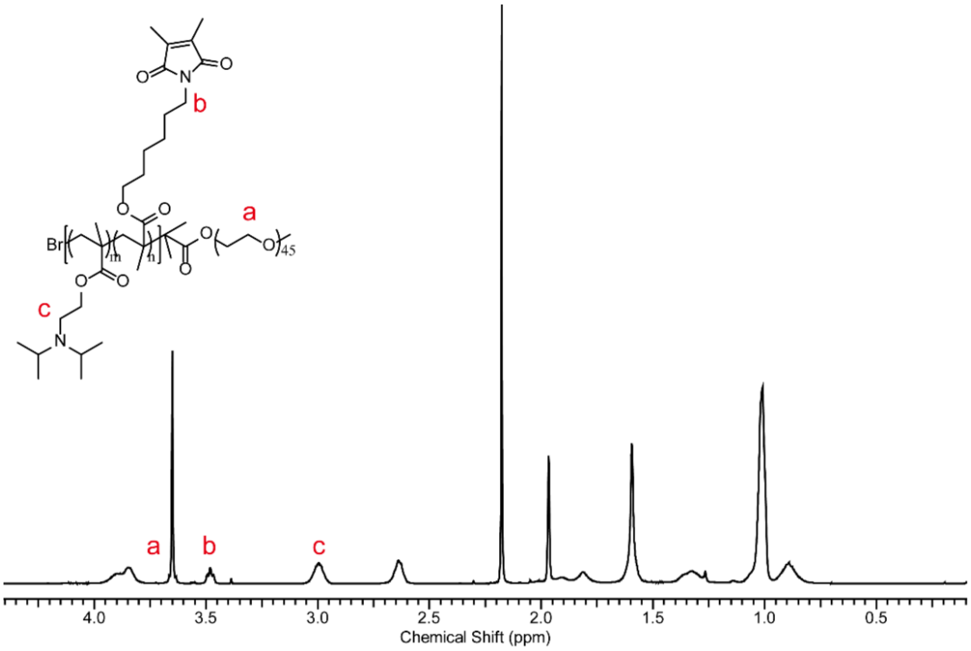

Figure S5. ^{1}H NMR spectrum of BCP-B for determining the final structure. The corresponding atoms are marked in the polymer structure.

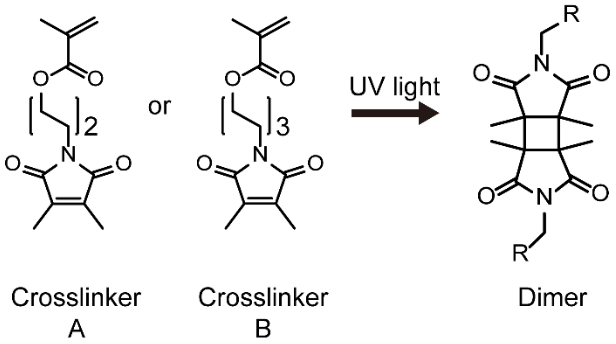

Figure S6. The chemical structures of photo-crosslinker A and B, and the crosslinking reaction for dimer forming.

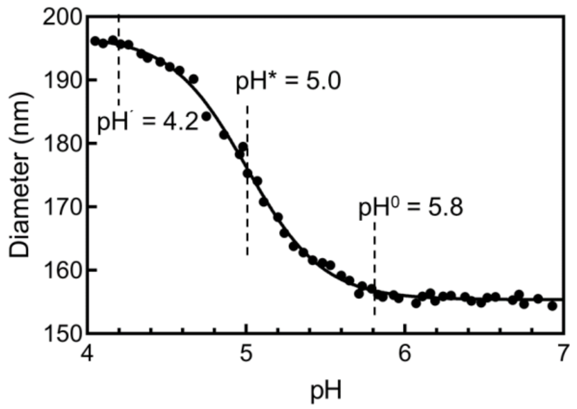

Figure S7. The schematic figure for calculating starting point of swelling (pH^0), ending point of swelling (pH´) and turning point of pH-dependent size transition (pH*).

A short explanation: Starting point of swelling (pH^0) is the pH value at which the size of polymersome start to increase indicating the start of swelling as pH decreases. Ending point of swelling (pH´) is the pH value at which the size of polymersome stop increasing indicating the end of swelling. When pH is lower than pH´, the size of polymersomes is uniform. Turning point of pH-dependent size transition (pH*) is calculated by the average of pH^0 and pH´. pH* = (pH^0 + pH´)/2.

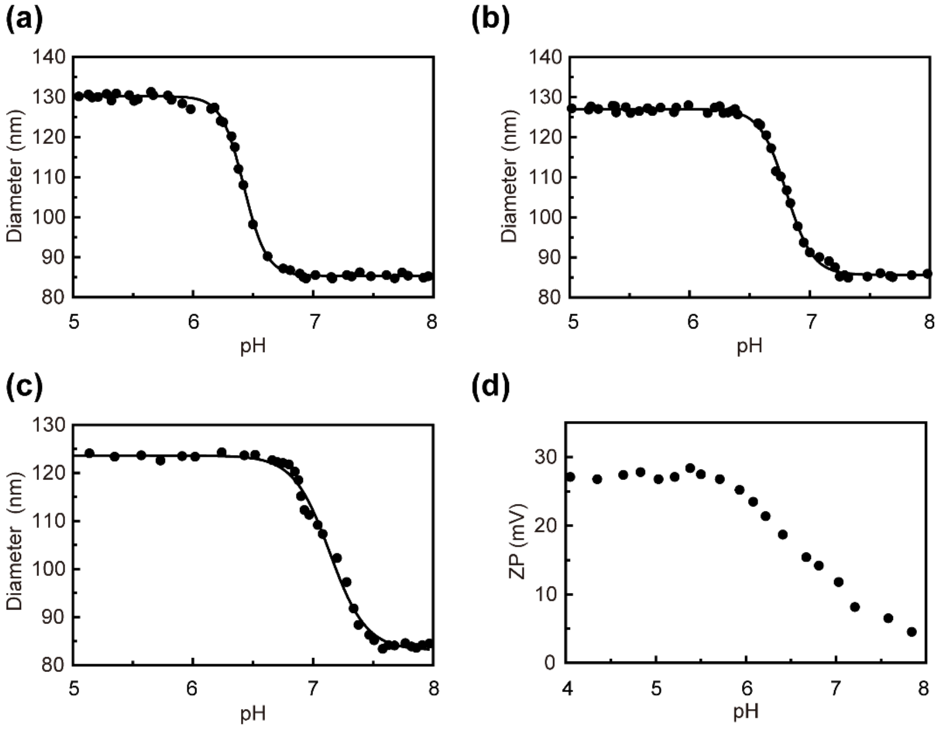

Figure S8. DLS titration data of Psome A in 10 mM NaCl solution (a), 1 mM PBS buffer solution (b) and 10 mM PBS buffer solution (c) (• Diameter from DLS, — Logistic fit of the curve). (d) Zeta potential curves of Psome A in 1 mM PBS buffer at various pH values.

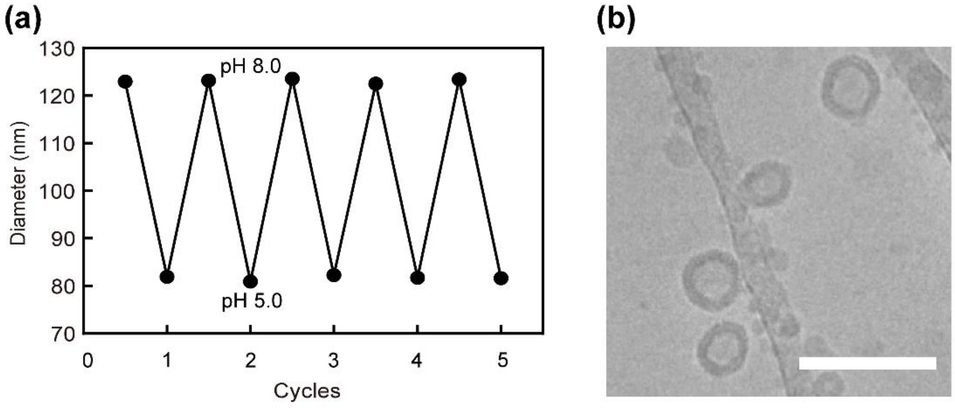

Figure S9. (a) Reversible swelling/deswelling of Psome A in water at pH 5.0 and 8.0 by DLS. (b) Cryo-TEM micrograph of Psome A, scale bar 200nm. (Diameter Ø = 80.4 ± 17.2 nm, membrane thickness M = 16.1 ± 1.7 nm, measured from above 100 Psome A).

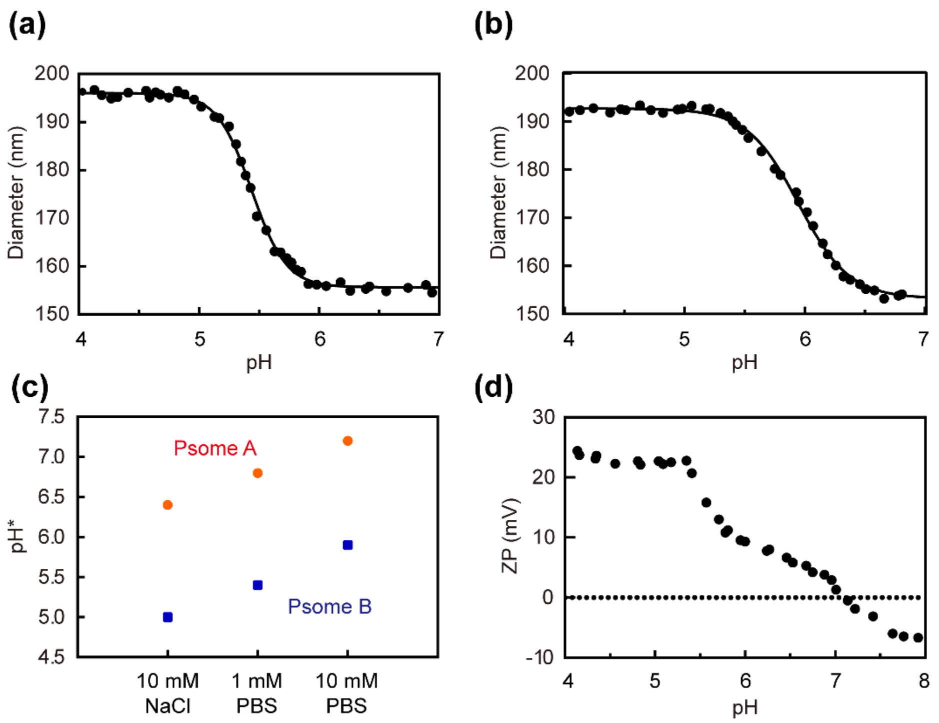

Figure S10. DLS titration data of Psome B in 1 mM PBS buffer solution (a) and 10 mM PBS buffer solution (b) (● Diameter from DLS, ━ Logistic fit of the curve). (c) The pH* of Psome A and B in 10 mM NaCl solution, 1 mM and 10 mM PBS buffer solution, separately. (d) Zeta potential curves of Psome B in 1 mM PBS buffer at various pH values.

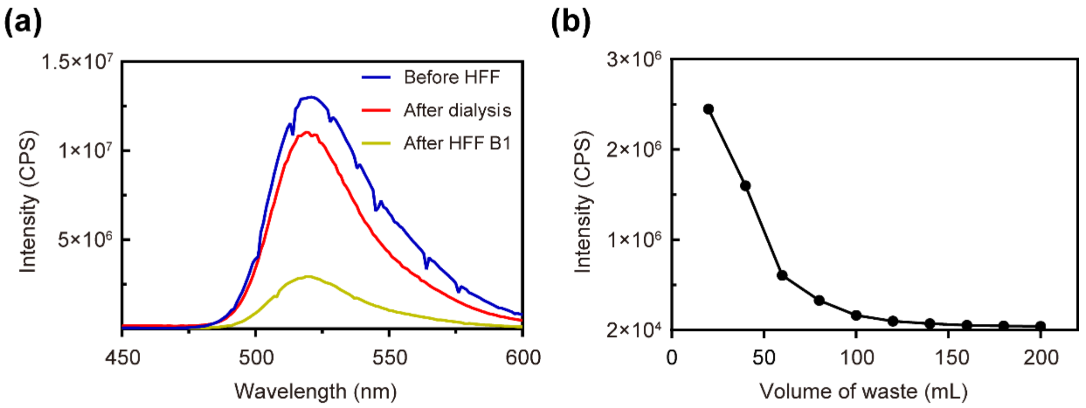

Figure S11. (a) The fluorescence spectra of avidin-Alexa Fluor 488-Psome B solution before and after purification by HFF 1 with 1 mM PBS buffer at pH 7.0 ($\lambda_{excitation}$ = 317 nm). (b) The fluorescence intensity of free avidin-Alexa Fluor 488 in the waste solution during the process of HFF 1 with 1 mM PBS buffer at pH 7.0 ($\lambda_{excitation}$ = 317 nm, $\lambda_{emission}$ = 518 nm).

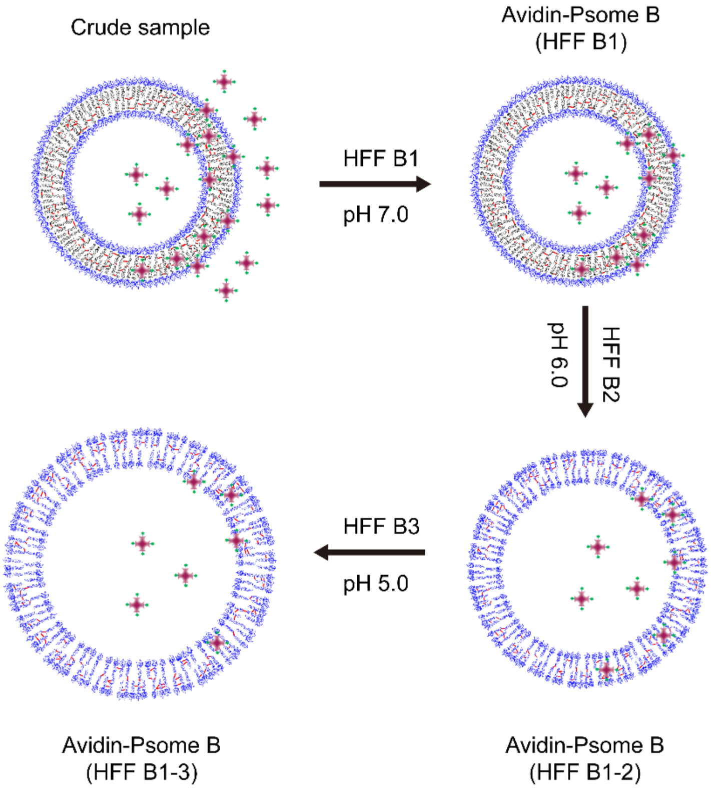

Figure S12. Protocol for the establishment of two-dimensional pH-stable avidin-Alexa Flour 488 loaded polymersome B (Avidin-Alexa Fluor 488-Psome B, AAF-Psome B) through sequential pH-dependent shear-force driven hollow-fiber-filtration (HFF).

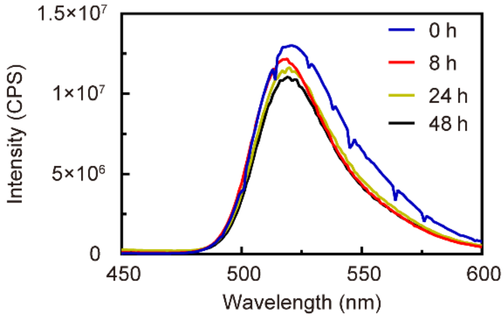

Figure S13. The fluorescence spectra of Psome B with in-situ loaded avidin-Alexa Flour 488 before dialysis and after dialysis against 1 mM PBS buffer at pH 7.0 for 8 h, 24 h and 48 h, separately.

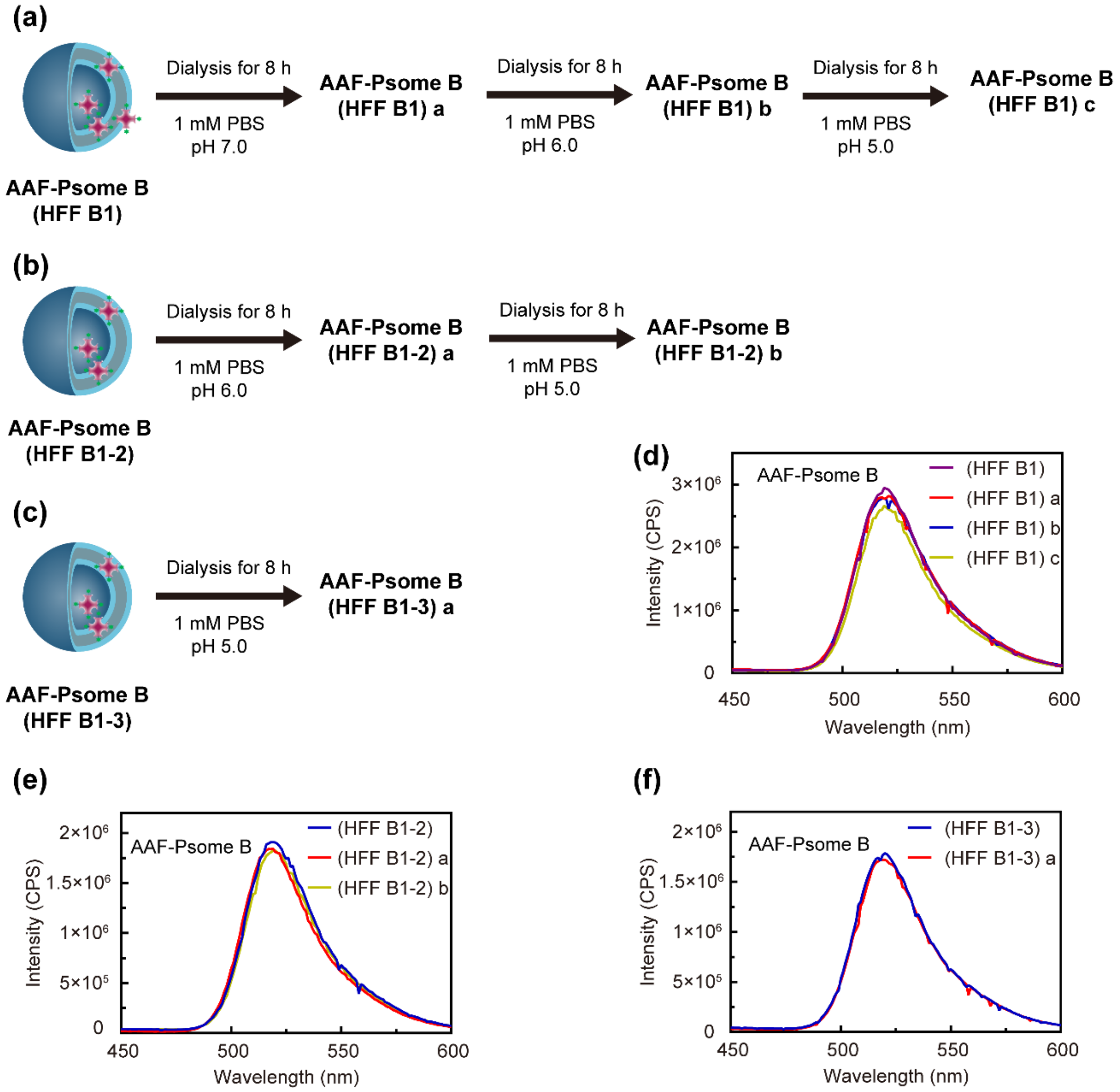

Figure S14. The protocol of purification of Avidin-Alexa Flour 488-Psome B by sequential dialysis (a, b, c) and the fluorescence spectra of AAF-Psome B (HFF B1), AAF-Psome B (HFF B1) a, AAF-Psome B (HFF B1) b and AAF-Psome B (HFF B1) c (d), AAF-Psome B (HFF B1-2), AAF-Psome B (HFF B1-2) a and AAF-Psome B (HFF B1-2) b (e), AAF-Psome B (HFF B1-3) and AAF-Psome B (HFF B1-3) a (f).

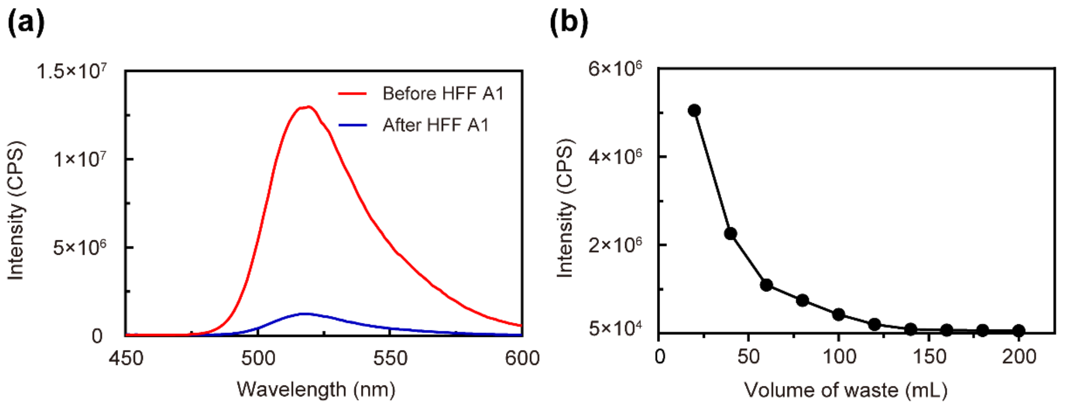

Figure S15. (a) The fluorescence spectra of avidin-Alexa Fluor 488-Psome A solution before and after purification by HFF A1 with 1 mM PBS buffer at pH 8.0 ($\lambda_{excitation}$ = 317 nm). (b) The fluorescence intensity of free avidin-Alexa Fluor 488 in the waste solution during the process of HFF A1 with 1 mM PBS buffer at pH 8.0 ($\lambda_{excitation}$ = 317 nm, $\lambda_{emission}$ = 518 nm).

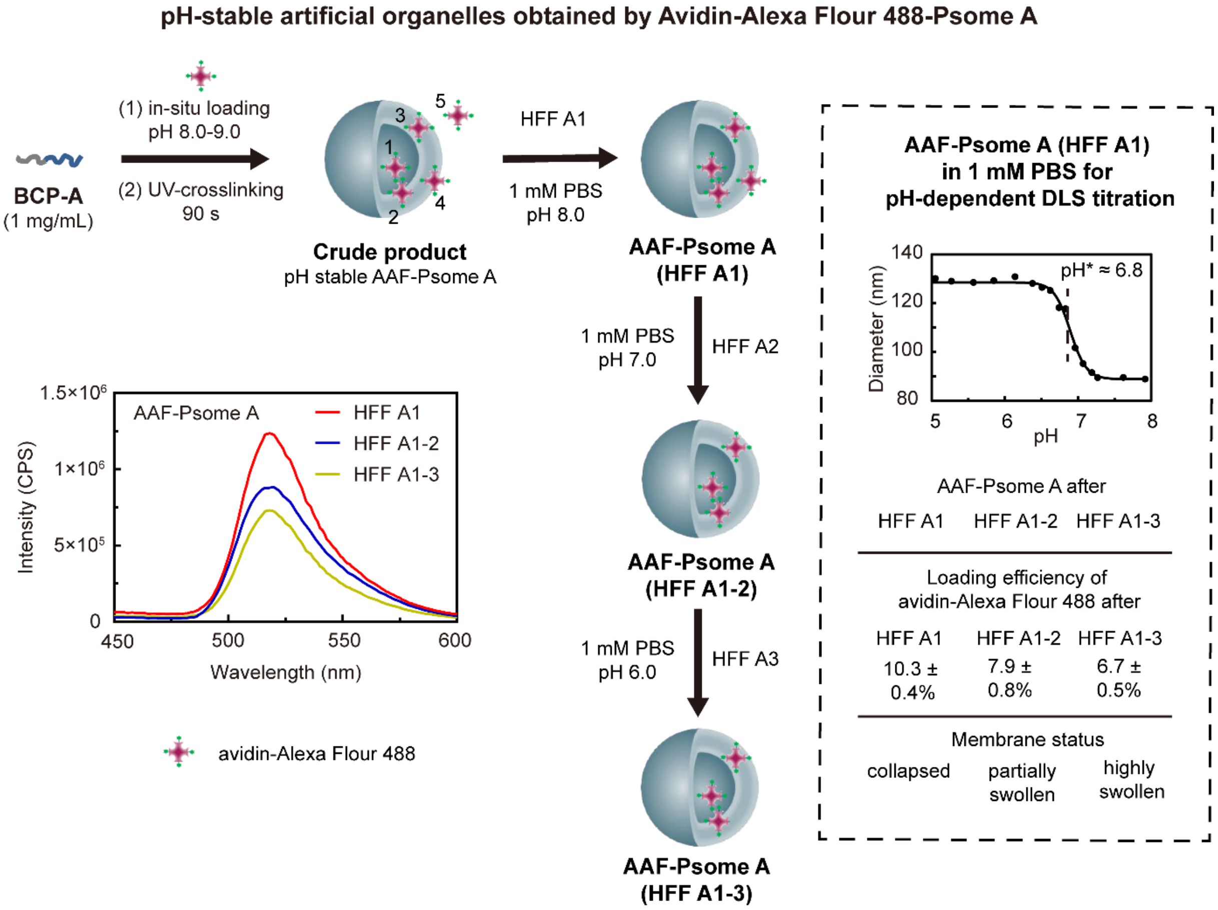

Figure S16. Protocol for the establishment of pH-stable avidin-Alexa Flour 488 loaded Psome A (Avidin-Alexa Flour 488-Psome A, AAF-Psome A) through sequential pH-dependent HFF. Loading efficiency (%) of AAF-Psome A after different HFF processes (right bottom) calculated from fluorescence spectra of all the samples (left). pH-dependent DLS titration of Avidin-Psome A (HFF A1) in 1 mM PBS (right) for validating collapsed membrane, partially swollen membrane and highly swollen membrane.

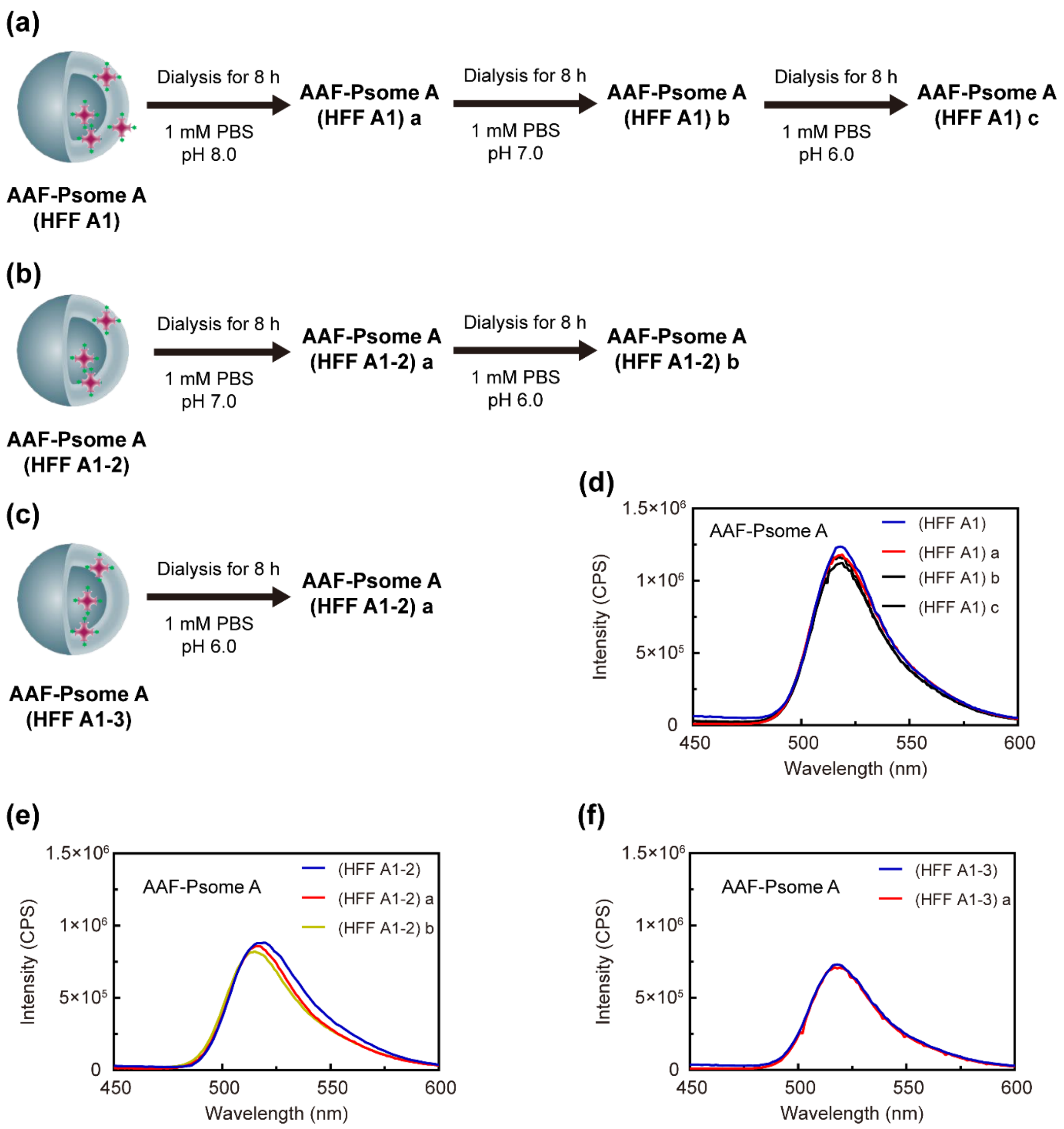

Figure S17. The protocol of purification of Avidin-Alexa Flour 488-Psome A by sequential dialysis (a, b, c) and the fluorescence spectra of Sample A1, A1a, A1b and A1c (d), Sample A2, A2a and A2b (e), Sample A3 and A3a (f).

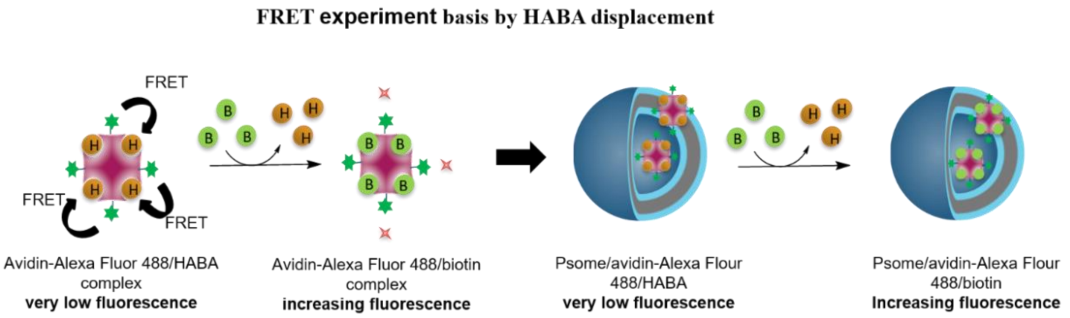

Figure S18. Schematic representation of FRET experiment basis by HABA displacement.

A short interpretation for the FRET basis: The complex of avidin-Alexa Fluor 488 conjugate and HABA, shows low fluorescence intensity via the FRET process. Once adding biotin, HABA is displaced by biotin which binds to the avidin-Alexa Fluor 488 conjugate, resulting in an increase of fluorescence intensity.

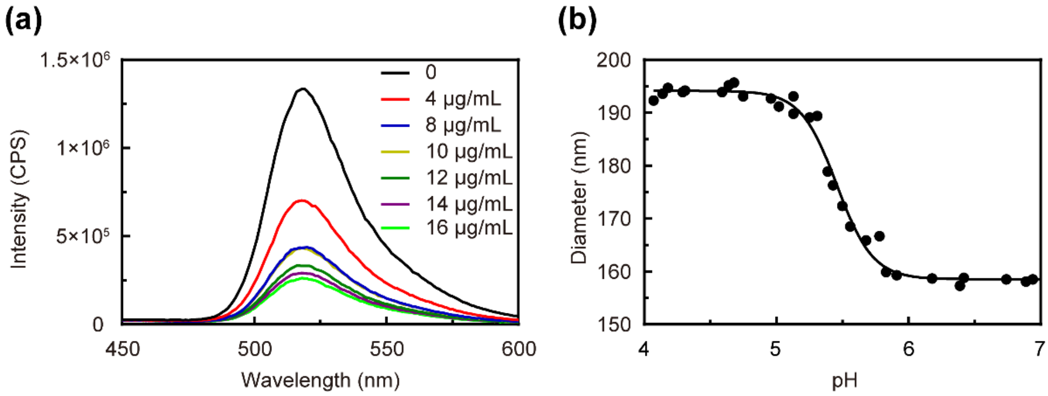

Figure S19. (a) The fluorescence spectra of AAF-Psome B (HFF B1) after adding different amount of HABA at pH 5.0. (b) DLS titration data of AAF-Psome B (HFF B1) in 1 mM PBS buffer solution in presence of 16 μg/mL HABA.

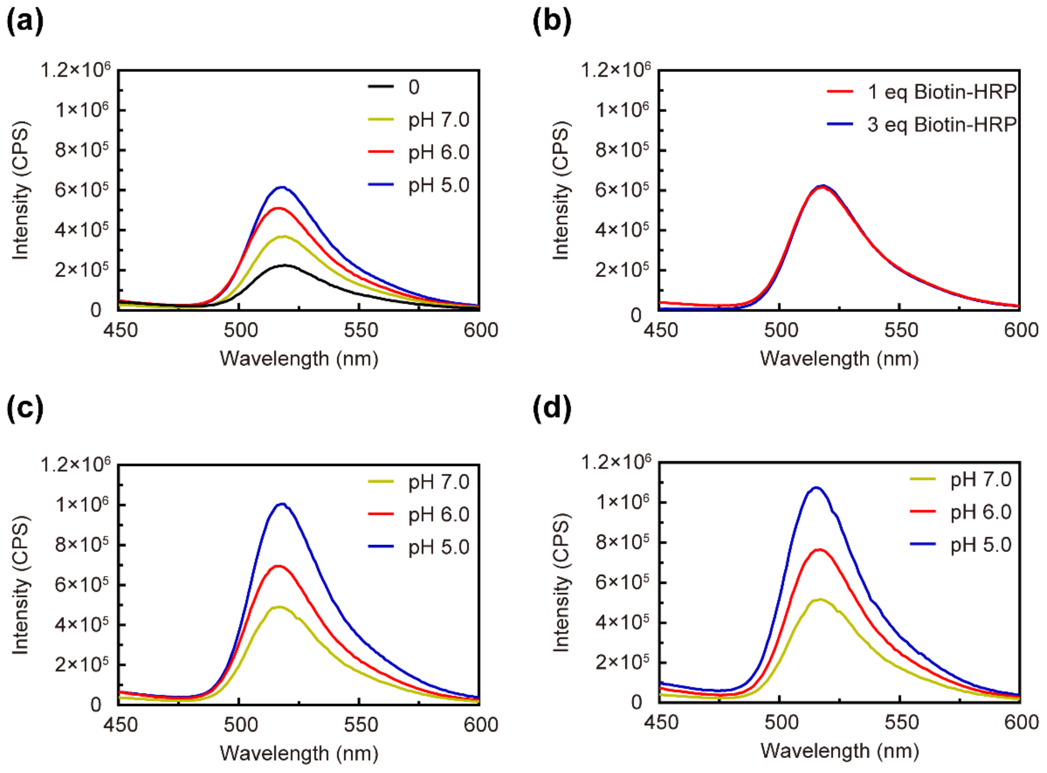

Figure S20. The whole fluorescence spectra of samples in Figure 4. (a) The fluorescence spectra of HAAP B (HRP) at pH 5.0 after adding 25 μg/mL Biotin-HRP solution at different pH. (b) The fluorescence spectra of HAAP B (HRP) at pH 5.0 after adding 25 μg/mL and 75 μg/mL Biotin-HRP solution at pH 5.0, respectively. (c) The fluorescence spectra of HAAP B (HRP + PEG) at pH 5.0 after adding 1.1 μg/mL Biotin-PEG_{3kDa} solution at different pH. (d) The fluorescence spectra of HAAP B (PEG) at pH 5.0 after adding 2.2 μg/mL Biotin-PEG_{3kDa} solution at different pH.

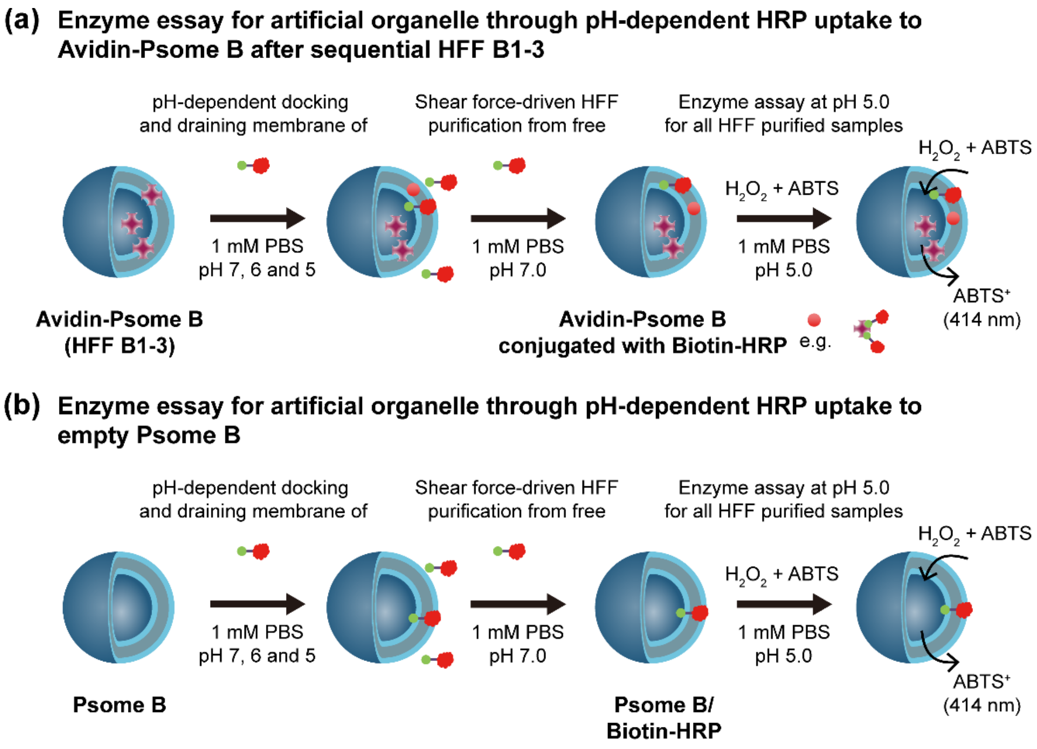

Figure S21. The protocol for enzyme assay of Biotin-HRP uptake to Avidin-Psome B (HFF B1-3) (a) and empty Psome B (b) after adding Biotin-HRP and HFF purification. (The control experiment of Figure 5)

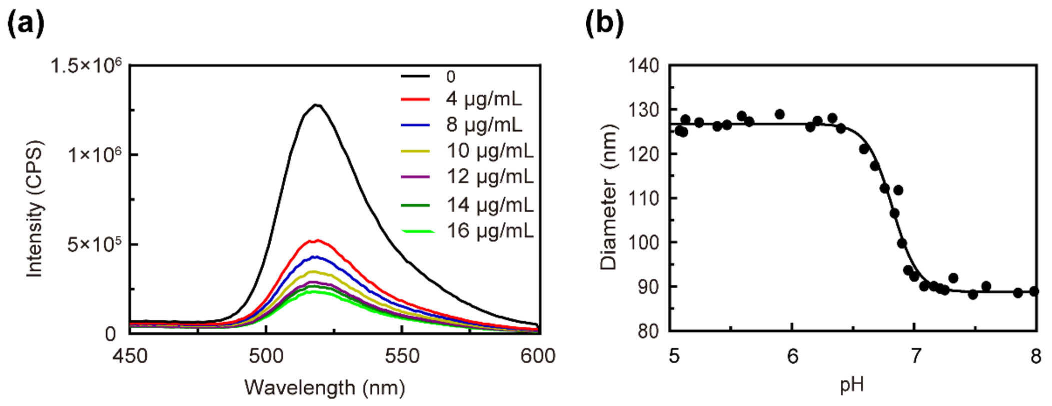

Figure S22. (a) The fluorescence spectra of AAF-Psome A (HFF A1) after adding different amount of HABA at pH 5.0. (b) DLS titration data of AAF-Psome A (HFF A1) in 1 mM PBS buffer solution in presence of 16 μg/mL HABA.

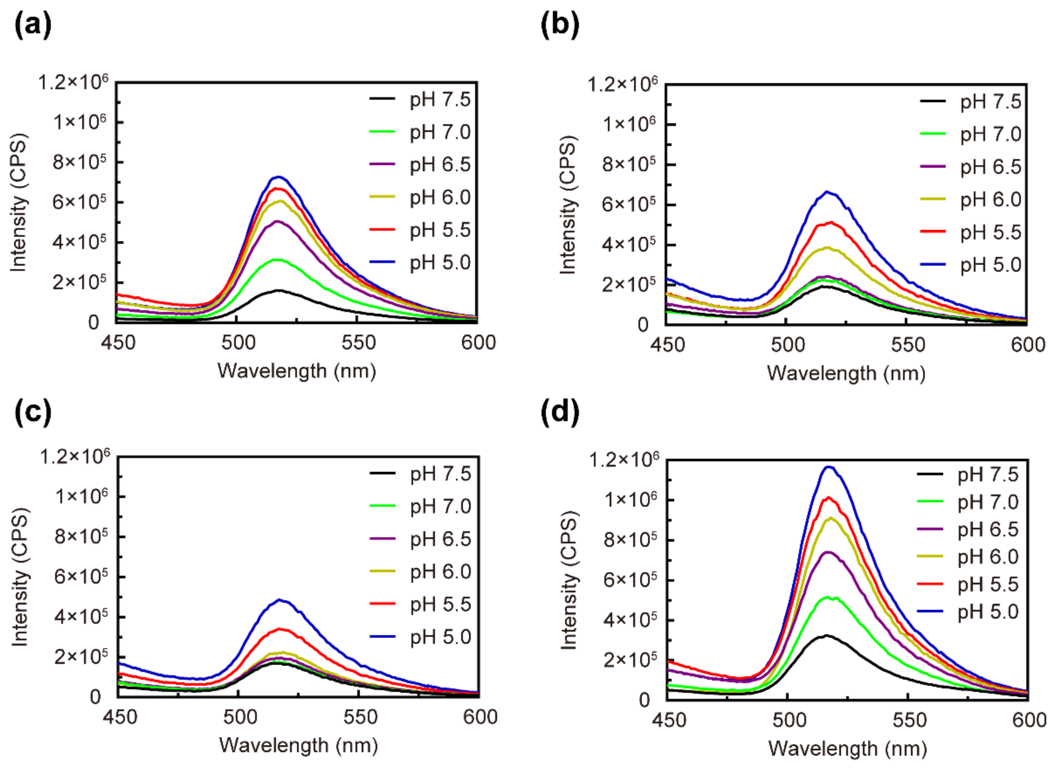

Figure S23. The whole fluorescence spectra of samples in Figure 7 (bottom). (a) The fluorescence spectra of Mixture 1 (HAAP-Psome A (HFF A1) and empty Psome B-HABA mixture) at pH 5.0 after adding 2.2 μg/mL Biotin-PEG_{3kDa} solution at different pH. (b) The fluorescence spectra of Mixture 2 (HAAP-Psome B (HFF B1) and empty Psome A-HABA

mixture) at pH 5.0 after adding 2.2 μg/mL Biotin-PEG_{3kDa} solution at different pH. (c) The fluorescence spectra of Mixture 3 (HAAP-Psome B (HFF B1-3) and empty Psome A-HABA mixture) at pH 5.0 after adding 2.2 μg/mL Biotin-PEG_{3kDa} solution at different pH. (d) The fluorescence spectra of Mixture 4 (HAAP-Psome A (HFF A1) and HAAP-Psome B (HFF B1) mixture) at pH 5.0 after adding 2.2 μg/mL Biotin-PEG_{3kDa} solution at different pH.

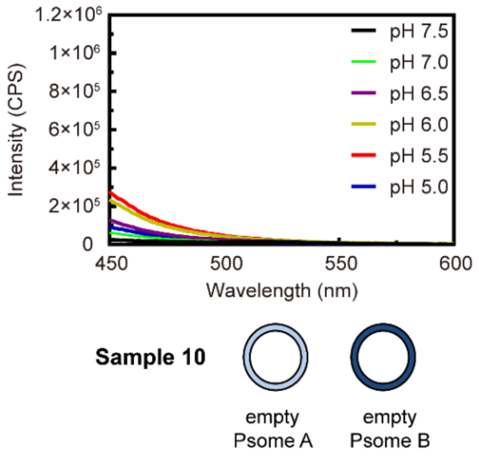

Figure S24. The fluorescence spectra of Mixture 5 (empty HABA-Psome A and B mixture) at pH 5.0 after adding 2.2 μg/mL Biotin-PEG_{3kDa} solution at different pH values.

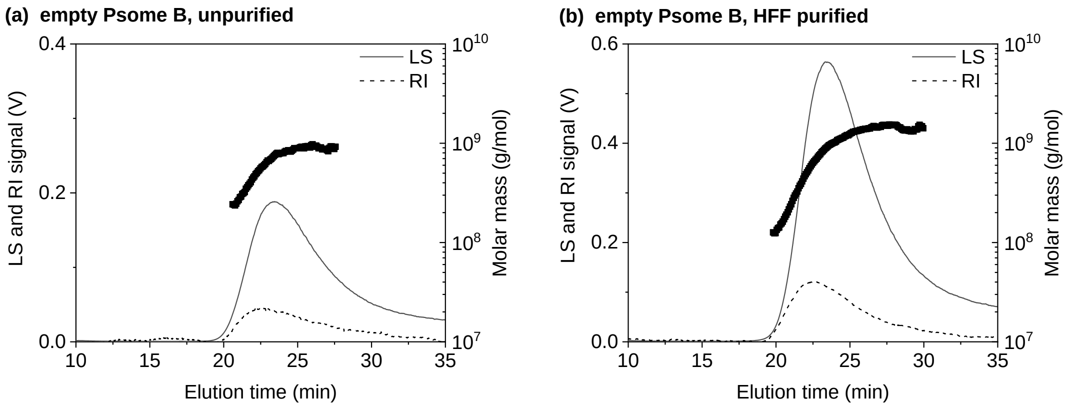

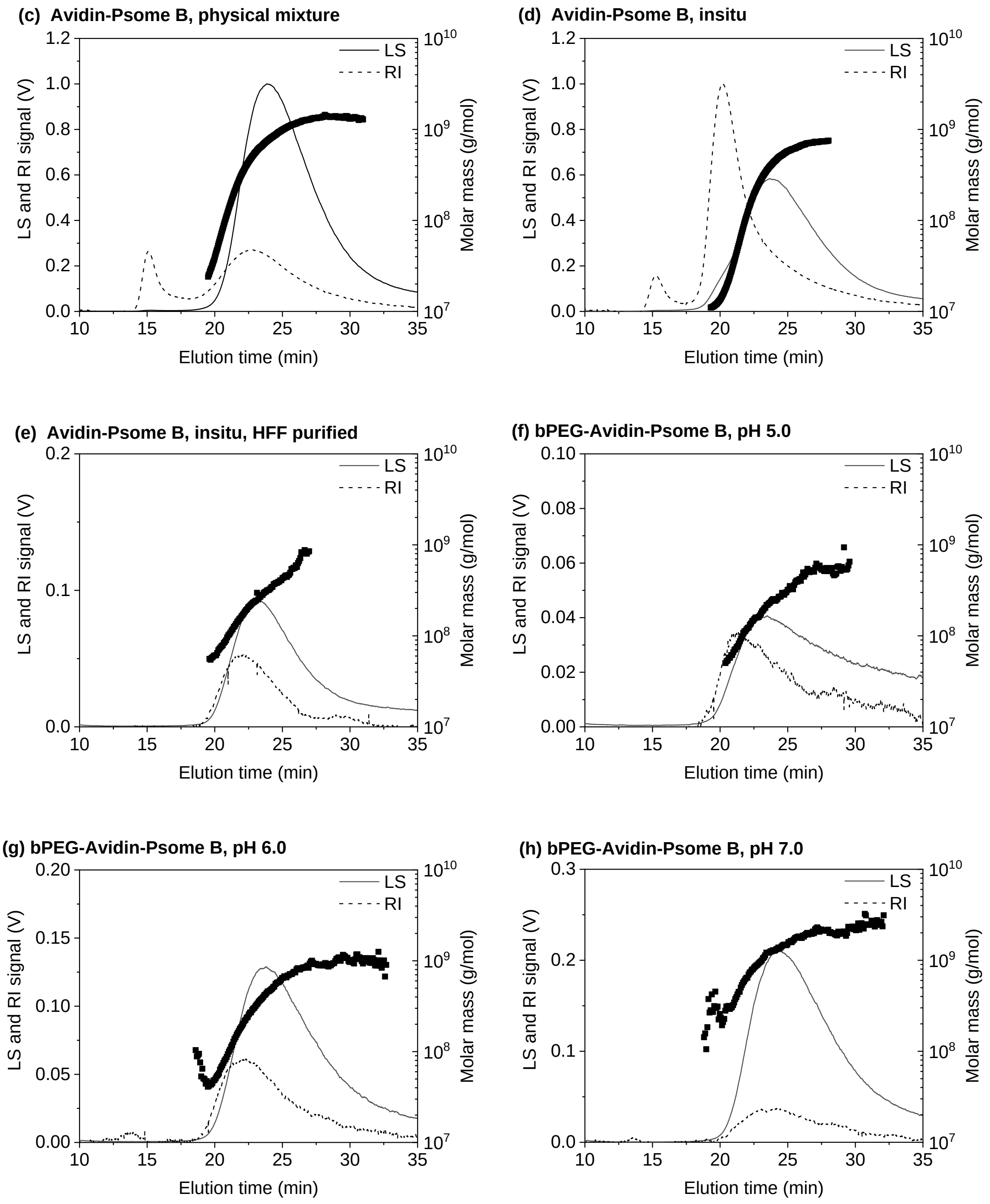

Figure S25. AF4 fractograms of different Psome B samples, LS and RI signals, and molar masses vs elution time.

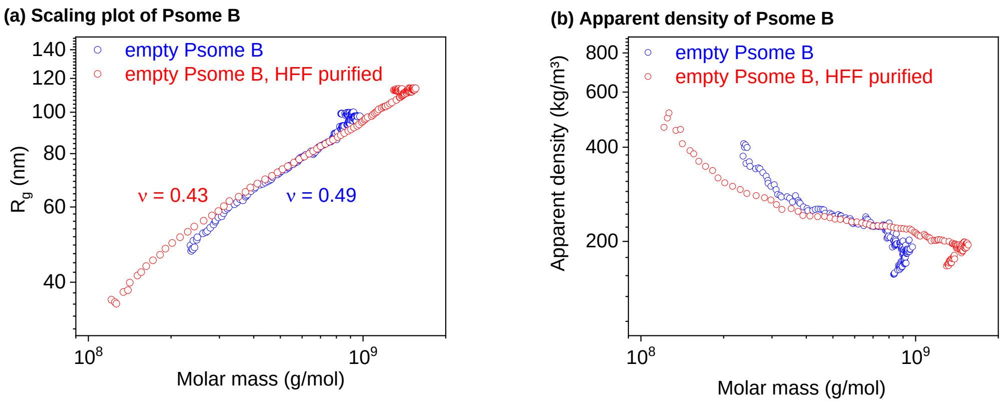

Figure S26. (a) Scaling plots, R_g vs. molar masses and (b) apparent densities vs. molar masses of empty Psome B before and after HFF purification samples determined by AF4.

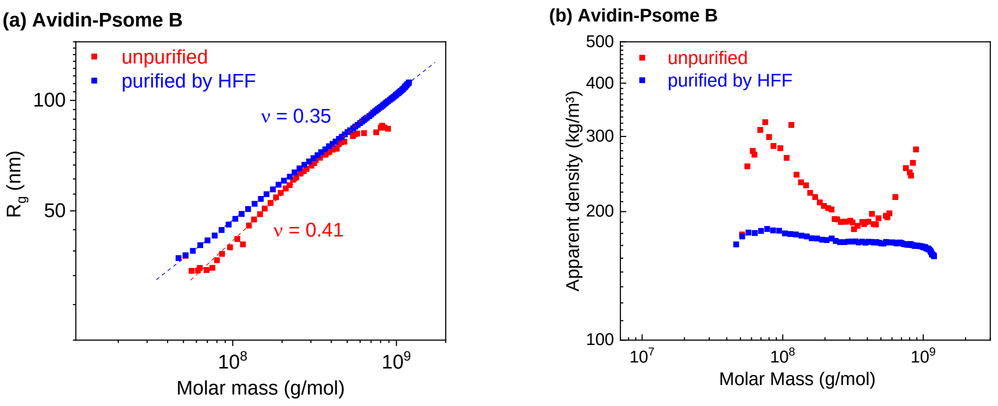

Figure S27. (a) Scaling plots, R_g vs. molar masses and (b) apparent densities vs. molar masses of Avidin-Psome B before and after HFF purification determined by AF4.

Data interpretation: The in-situ loading process of Avidin influences the formation of Psome B. Compared to the Psome B without Avidin, the molar masses are lower and radii are higher due to integration of Avidin. In case of in-situ loaded Avidin-Psome B, a certain free amount is detected in RI signal due Avidin excess (Figure S25d). Obviously, a certain amount of Avidin biomacromolecules are located on the surface of the membrane. After the purification of Avidin-Psome B by HFF, the free Avidin biomacromolecules in the solution and from the

membrane surface are removed (Figure S25e) and a decrease of the scaling parameter ν from 0.41 to 0.35 is observed (Figure S27a). The conformation changes from irregular, compact shape to more spherical architecture after the removal of Avidin biomacromolecules from the membrane surface. Furthermore, the decrease of density can be explained with the purification step.

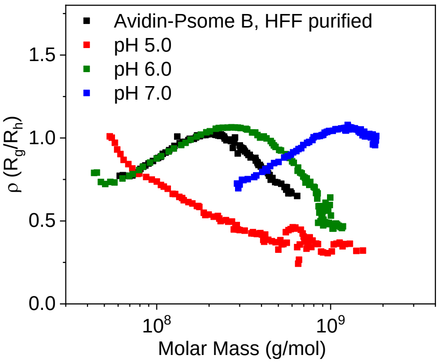

Figure S28. ρ parameter (R_g/R_h) vs molar masses of Avidin-Psome B, HFF purified with different Biotin-PEG_{3kDa} loading conditions (pH 5.0…red; pH 6.0…green; pH 7.0…blue) determined by AF4.

7. References

[1] J. Gaitzsch, D. Appelhans, L. Wang, G. Battaglia, B. Voit, *Angew. Chem. Int. Ed.* **2012**, *51*, 4448.

[2] D. Gräfe, J. Gaitzsch, D. Appelhans, B. Voit, *Nanoscale* **2014**, *6*, 10752.

[3] W. Burchard, *Adv. Polym. Sci.* **1999**, *143*, 113.

[4] S. Moreno, S. Boye, A. Lederer, A. Falanga, S. Galdiero, S. Lecommandoux, B. Voit, D. Appelhans, Biomacromolecules **2020**, *21*, 5162.
